# Terminal acceptor engineering for reduced energy dissipation and enhanced charge transport in benzodithiophene-core based donor molecules: a computational route to efficient organic solar cells

**DOI:** 10.1039/d5na01002k

**Published:** 2026-04-23

**Authors:** Sabiha Khanam, Zahraa Falah Khudair, Ines Hilali Jaghdam, Rana Farhat Mehmood, Muhammad Imran, Mohamed S. Soliman, Ahmed M. Shawky, Rabaa Bousbih, Syed Muhammad Kazim Abbas Naqvi, Rasheed Ahmad Khera

**Affiliations:** a Department of Chemistry, University of Agriculture Faisalabad 38000 Pakistan; b Department of Chemistry, College of Education, University of Al-Qadisiyah Iraq; c Department of Computer Science and Information Technology, Applied College, Princess Nourah bint Abdulrahman University P.O. Box 84428 Riyadh 11671 Saudi Arabia; d Department of Chemistry, University of Education DG Khan Campus Lahore Pakistan; e Department of Chemistry, Faculty of Science, Research Center for Advanced Materials Science (RCAMS), King Khalid University P.O. Box 960 Abha 61421 Saudi Arabia; f Department of Electrical Engineering, College of Engineering, Taif University Taif 21944 Saudi Arabia; g Department of Chemistry, Faculty of Science, Umm Al-Qura University 21955 Makkah Saudi Arabia; h Department of General Studies, University of Prince Muqrin Medina 42361 Saudi Arabia; i Physics department, Faculty of Science, University of Tunis ElManar Tunis 1060 Tunisia; j Faculty of Materials Science, Shenzhen MSU-BIT University Shenzhen 518115 China; k Platform for Applied Nanophotonics, Institute of Advanced Interdisciplinary Technology, Shenzhen MSU-BIT University Shenzhen 518115 China

## Abstract

The development of donor materials with the finely tuned frontier orbital alignment, broad optical absorption and efficient charge transport is still a key challenging issue for next generation high efficiency organic solar cells (OSCs). In this study, benzodithiophene-centered small donor molecules were systematically designed *via* terminal acceptor modification to optimize optical, electronic, and photovoltaic properties. Density functional theory (DFT) and time dependent DFT (TD-DFT) calculations at the B3LYP/6-31G(d,p) level reveal that strategic substitution with strongly electron withdrawing terminals induces substantial intramolecular charge transfer (ICT), narrows the bandgap (*E*_g_) and red shifts the absorption maxima up to 903 nm in solvent. Density of states and transition density matrix calculations reveal efficient charge delocalization from donor to acceptor, especially for SM-6 and SM-8, which have larger off-diagonal electron–hole coupling and planarized backbones that are favorable for π–π stacking. The exciton binding energies (*E*_b_) decrease from 0.26 to 0.14 eV, whereas the electron reorganization energy (*λ*_e_) goes down from 0.178 to 0.091 eV, indicating improved charge mobility and reduced energetic disorder. Theoretical device level parameters show enhanced open circuit voltages (*V*_oc_) up to 1.39 eV, high fill factors (FF) approaching 0.91, and minimized energy losses (*E*_loss_) down to 0.42 eV for the optimized donors. Collectively, these results demonstrate that terminal group engineering in BDT-based donors effectively tailors energy level alignment, light harvesting, and charge transport, establishing a rational design pathway toward small molecule donor systems capable of achieving high photovoltaic efficiency in OSCs, particularly through the simultaneous reduction of electron reorganization energy and energy loss.

## Abstract 2

Optimized molecules show red shifted absorption, stronger delocalization, lower exciton binding energy, and improved π–π stacking.

## Graphical abstract

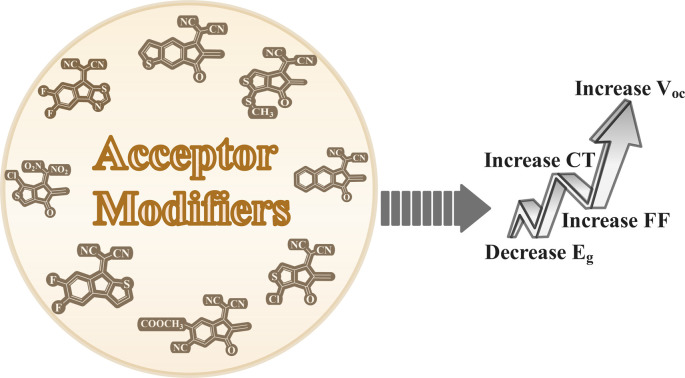

## Introduction

Progressive advances in donor–acceptor (D–A) materials, combined with precise control over bulk heterojunction (BHJ) morphology, have increased single junction organic solar cell (OSC) efficiencies above 20%.^1–3^ Owing to their solution processability, mechanical flexibility and tunable optical properties, OSCs are suitable for scalable and large area fabrication of lightweight, flexible, and semi-transparent organic photovoltaic (OPV) devices.^4–6^ Early in the development of OPV technology, fullerene derivatives such as PC_61_BM and PC_71_BM were the predominant electron acceptors due to their high electron affinity and favourable phase compatibility with conjugated donors.^7,8^ Despite achieving power conversion efficiencies (PCEs) exceeding 10%, fullerene-based blends faced limitations due to their fixed LUMO level, poor morphological stability, and low photo absorption properties in the visible and near-infrared (NIR) regions.^9–11^ The emergence of fused-ring non-fullerene acceptors (NFAs) transformed the field by enabling broader absorption spectra (*λ*_max_), tunable bandgap (*E*_g_), and improved morphological stability.^12^ Collectively, these characteristics established a new era of high efficiency OSCs. With a broad *λ*_max_ and high electron mobility, ITIC (A–D–A acceptor) enabled PBDB-T based OSCs to reach improved PCEs.^12^ The fluorinated derivative IT-4F further red shifted *λ*_max_ and lowered the LUMO, leading to a PCE of 14.7% with the copolymer donor PTO2.^13^ Additionally, Y-series acceptors, extended *λ*_max_ to almost 900 nm, reduced the non-radiative energy loss pathways, and enabled single junction OSCs to exceed 20% PCE.^14^ With the rapid development of NFAs, research is increasingly focused on designing donor materials that complement their broadened *λ*_max_ and finely tuned *E*_g_ to enable further improvements in the performance of OSCs.^15,16^

Benzodithiophene (BDT)-based donors have shown excellent potential to meet these requirements. Their planar fused-ring core structure, strong electron donating character, high thermal stability, and tendency toward π–π stacking facilitate high hole mobility and efficient charge extraction.^17–19^ Structural optimization has progressively enhanced device efficiencies. The PBDB-T : ITIC blend achieved a PCE of 11.2%, which increased to 13.1% when PBDB-T-SF was employed as the donor with IT-4F as the acceptor.^20^ The di-fluorinated derivative PM6 combined with Y6 attained 15.7% PCE by suppressing non-radiative energy loss to approximately 0.54 eV.^21,22^ Moreover, chlorinated PM7 with Y6 delivered 17.0% PCE, attributed to its deeper HOMO level, which raised open circuit voltage (*V*_oc_) to around 0.90 V.^23,24^ These results indicate that π-conjugation extension, side chain engineering, and particularly terminal group engineering critically influence the *E*_g_, exciton binding energy (*E*_b_), molecular packing, and BHJ morphology. While polymeric BDT donors dominate current high efficiency OSCs, small molecule donors (SMDs) remain attractive due to their distinct molecular weights, high chemical purity, and consistent molecular packing. These characteristics promote balanced charge transport, stable BHJ morphologies, and reduced energetic losses.^25^ Early SMD:PCBM devices yielded modest PCEs around 7.4%.^26,27^ However, subsequent molecular refinements, including π-bridge extension, halogen substitution, and terminal group engineering, have enhanced intramolecular charge transfer (ICT), red shifted *λ*_max_, and improved hole mobility, significantly narrowing the performance gap with polymer based OSCs.^28,29^ Collectively, these developments highlight terminal acceptor engineering as a powerful route to tailor SMD optoelectronic properties for high-performance OSCs.

In this study, density functional theory (DFT) and time-dependent DFT (TD-DFT) calculations were employed to examine the optoelectronic properties of benzodithiophene-centered SMDs for OSCs. This work is intended as a predictive computational design study aimed at guiding experimental synthesis and device optimization of benzodithiophene-based small molecule donors for OSCs. We designed a series of eight derivatives (SM-1 to SM-8) based on a reference donor molecule SM-R (ref. 30) to explore the role of terminal acceptor engineering in BDT-centered SMDs. Each designed molecule contains a BDT core connected to its end groups through a fluorinated thiophene π-bridge. The terminal acceptor groups were systematically varied aiming to tune *E*_g_, *λ*_max_ and ICT strength. The results show that terminal acceptor engineering red shifts *λ*_max_, downshifts the HOMO level to improve *V*_oc_, lowers the *E*_b_, and strengthens π–π stacking, thereby improving charge transport. Overall, the designed donor molecules exhibit optimized optoelectronic characteristics, highlighting their potential for next generation OSCs.

## Theoretical methods

All quantum chemical calculations were performed using DFT as implemented in the Gaussian 09 package. Geometry optimizations of the reference molecule SM-R and its derivatives were carried out, and benchmarking with several hybrid functionals identified B3LYP/6-31G as the most reliable level of theory. This functional yielded absorption maxima in closest agreement with experimental data and was therefore employed for all subsequent calculations. Time-dependent self-consistent field (TD-SCF) simulations in the gaseous phase and chloroform (CHCl_3_) were conducted to evaluate the UV-visible absorption spectra. Molecular properties including binding energies (*E*_b_),^31^ open circuit voltage (*V*_oc_)^32^ and dipole moment (*D*)^33^ were computed to assess the charge distribution, interfacial compatibility, and photovoltaic (PV) potential of the designed molecules. Additional computational details, including functional benchmarking, density of states (DOS), transition density matrix (TDM)^34^ analysis, and reorganization energy (RE) calculations,^35,36^ are provided in the SI.

## Results and discussion

### Structural optimization

**Fig. 1:**
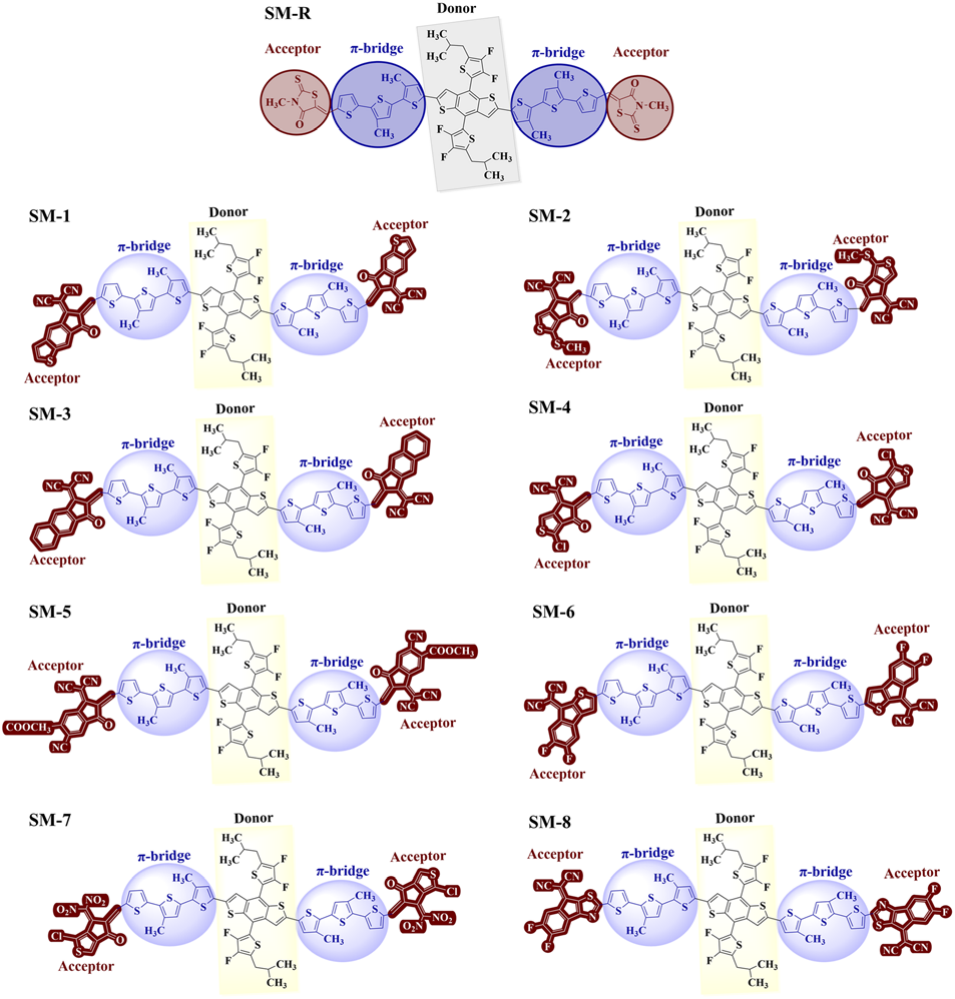
Structural design of the reference molecule and designed benzodithiophene-based donor molecules.

The reference small molecule SM-R is a symmetric A–π–D–π–A system in which thiazolidinone end groups act as terminal acceptors conjugated to a fluorinated, sulfur rich fused ring donor through thiophene π-bridges. To examine how the strength and size of the terminal acceptors influence molecular geometry, eight derivatives (SM-1 to SM-8) were designed by replacing the terminal end groups with a set of more electron withdrawing acceptors, including dicyanovinyl carbonyl and indanone based carbonyl acceptors, as well as chlorinated, ester substituted, difluoro substituted, nitro substituted, and N heteroaryl fluorinated variants. Fig. 1 shows the molecular segmentation of all the designed and reference molecule. All structures were optimized in the ground state at the B3LYP/6-31G(d,p) level prior to excited state analysis. This protocol provides reliable equilibrium geometries for assessing ICT tendencies and for extracting the structural descriptors reported in Table 1. The corresponding optimized geometries are shown in Fig. S2. The central C–C bond length *I*_c–c_ remains narrowly distributed from 1.41 to 1.43 Å across the series. These values lie between those of a typical single bond (about 1.54 Å) and a double bond (about 1.34 Å), indicating partial double bond character and effective conjugation along the backbone.^37,38^ The shorter values in SM-4, SM-5, and SM-7 are consistent with slightly enhanced bond order and delocalization near the donor–bridge junction, whereas SM-6 and SM-8 reproduce the SM-R metric, indicating that these substitutions preserve the reference bonding pattern.^39,40^

**Table 1:** Structural parameters of SM-R and designed molecules. Central C–C bond length (*I*_c–c_) and inter fragment dihedral angle (θ°) from ground state B3LYP/6-31G(d,p) optimizations

Molecule	Bond length (*I*_c–c_) (Å)	Dihedral angle (θ°)
SM-R	1.43	0.06
SM-1	1.42	12.77
SM-2	1.42	13.88
SM-3	1.42	12.00
SM-4	1.41	13.39
SM-5	1.41	11.72
SM-6	1.43	0.12
SM-7	1.41	8.84
SM-8	1.43	0.15

Backbone coplanarity, quantified by the inter-fragment dihedral angle θ, is more sensitive to substitution. Three molecules are effectively planar and therefore conducive to extended π overlap: SM-R (0.06°), SM-6 (0.12°), and SM-8 (0.15°). A second group shows moderate twisting: SM-7 (8.84°), SM-5 (11.72°), and SM-3 (12.00°). The largest twists occur for SM-1 (12.77°), SM-4 (13.39°), and SM-2 (13.88°), consistent with increased crowding from stronger or bulkier end groups, and with chlorine substitution in some cases at the D–A junction.^41^

Taken together, these results show that changing the terminal acceptors adjusts geometry primarily by altering backbone twisting rather than by disrupting the central bond order.^42^ The near planar set (SM-R, SM-6, and SM-8) should favor closer π–π contact, lower energetic disorder, and efficient through stack charge transport.^43,44^ The moderately twisted set (SM-3, SM-5, and SM-7) may accept a small reduction in π overlap while mitigating over aggregation and improving blend miscibility. The most twisted set (SM-1, SM-2, and SM-4) may exhibit weaker π stacking, but their stronger electron withdrawing end groups are expected to increase ICT character, narrow the *E*_g_, and assist exciton dissociation.^44,45^

### Frontier orbital energetics and alignment

**Fig. 2:**
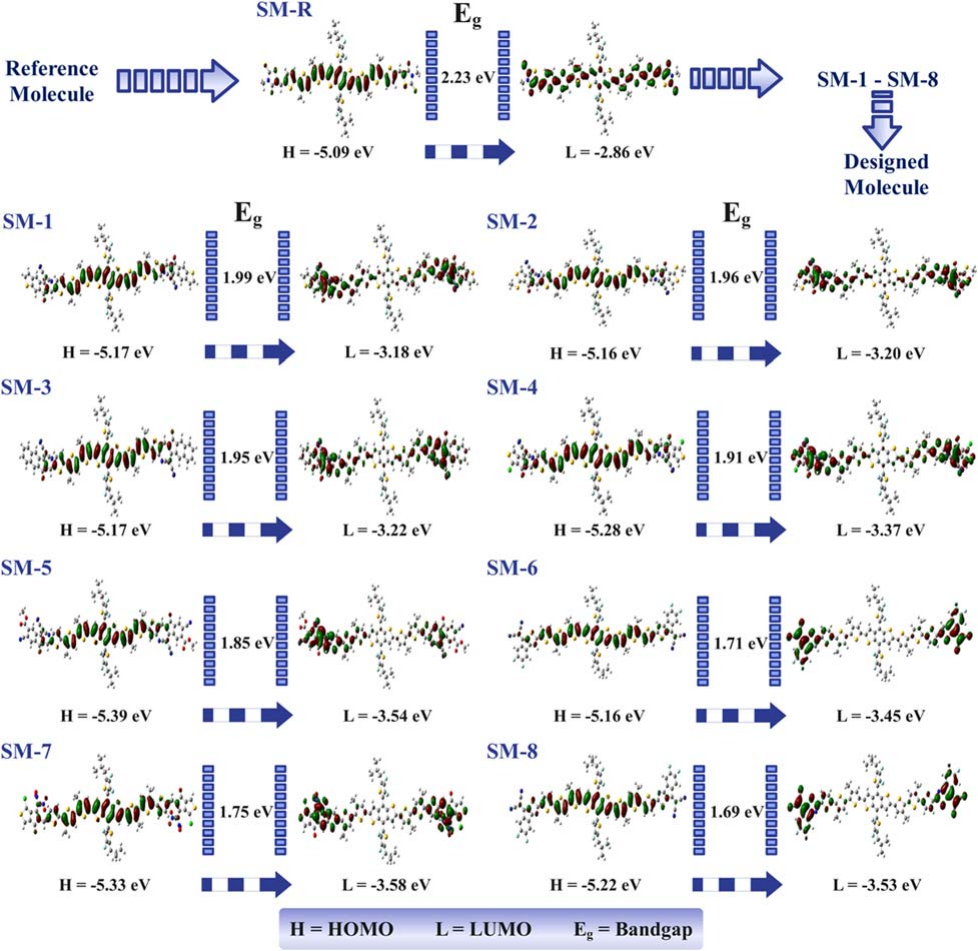
Frontier molecular orbitals and bandgap evolution of the reference and designed donor molecules.

Photocurrent (*J*_sc_) and *V*_oc_ in OSCs depends on the energy level offset between the donor HOMO and the acceptor LUMO, as well as the *E*_g_ and *λ*_max_ of the donor.^7,46^ To place each design within this balance, we evaluated *E*_HOMO_, *E*_LUMO_, and *E*_g_ for SM-R and the designed molecules, as shown in Fig. 2 and summarized in Table 2. End-group substitution adjusts the electronic structure through inductive and resonance effects and by extending conjugation onto the terminal units. Electron withdrawing terminals preferentially stabilize the π orbitals localized on the terminal units, which lowers the donor LUMO energy, whereas the donor HOMO, delocalized mainly on the fused central donor and thiophene bridges, shifts much less. Consequently the LUMO generally moves downward more than the HOMO, producing net *E*_g_ contraction.^11^ This modest HOMO variation reflects its localization on the fused central donor and π-bridges, making it less responsive to end-group substitution. The expected outcome is a larger downward shift of *E*_LUMO_ than of *E*_HOMO_ and a net contraction of *E*_g_.^47^ Relative to SM-R, all designed molecules show smaller *E*_g_.

**Table 2:** Electronic parameters (*E*_HOMO_, *E*_LUMO_, and *E*_g_) of SM-R and designed donor molecules

Molecule	*E*_HOMO_ (eV)	*E*_LUMO_ (eV)	*E*_g_ (eV)
SM-R	−5.09	−2.86	2.23
SM-1	−5.17	−3.18	1.99
SM-2	−5.16	−3.20	1.96
SM-3	−5.17	−3.22	1.95
SM-4	−5.28	−3.37	1.91
SM-5	−5.39	−3.54	1.85
SM-6	−5.16	−3.45	1.71
SM-7	−5.33	−3.58	1.75
SM-8	−5.22	−3.53	1.69

The individual values of *E*_g_ are 2.23 eV for SM-R, 1.99 eV for SM-1, 1.96 eV for SM-2, 1.95 eV for SM-3, 1.91 eV for SM-4, 1.85 eV for SM-5, 1.71 eV for SM-6, 1.75 eV for SM-7, and 1.69 eV for SM-8. Ordering *E*_g_ from smallest to largest gives SM-8 < SM-6 < SM-7 < SM-5 < SM-4 < SM-3 < SM-2 < SM-1 < SM-R. This order mirrors the increase in electron withdrawal at the molecular ends. While this identifies the LUMO as the primary lever for *E*_g_ reduction, the critically important result for a donor material is the relatively modest variation in the HOMO level (−5.09 eV to −5.39 eV). This indicates that the dramatic narrowing of the optical gap is achieved without a severe penalty to the ionization potential, which helps preserve *V*_oc_ when paired with common acceptors. Competing effects explain deviations within the simple trend. SM-7 has a very deep LUMO, yet its *E*_g_ is larger than SM-6 and SM-8 because the HOMO is also stabilized, which partly offsets LUMO lowering. Designs that remain essentially planar, such as SM-6 and SM-8, preserve conjugation and convert LUMO stabilization into stronger *E*_g_ compression. More twisted members, for example SM-1, SM-2, and SM-4, reduce through bond coupling and show less *E*_g_ contraction even with strong electron withdrawing end groups. The orbital maps in Fig. 2 show that the HOMO is primarily distributed on the fused donor core and π-bridges, while the LUMO is predominantly localized on the terminal electron withdrawing units. This spatial separation establishes a donor to acceptor charge transfer pathway and provides a microscopic rationale for the red shift of the lowest energy absorption band. Under Koopmans' approximation, stabilization of the LUMO translates into higher electron affinity, whereas limited HOMO shifts help preserve the ionization energy.^48^ From a device standpoint, very small *E*_g_ such as those of SM-8 and SM-6 maximize spectral coverage and can raise *J*_sc_, provided that the D–A offset remains ≥0.3 eV to enable efficient charge separation and that film morphology stays stable. Donors with an intermediate *E*_g_ such as SM-4 represent a strategic balance. Its narrower *E*_g_ compared to SM-R enhances *J*_sc_, while its HOMO level (−5.28 eV) is not as deeply stabilized as in SM-5 or SM-7, thus better preserving the *V*_oc_.^49,50^

### Molecular electrostatic potential analysis

**Fig. 3:**
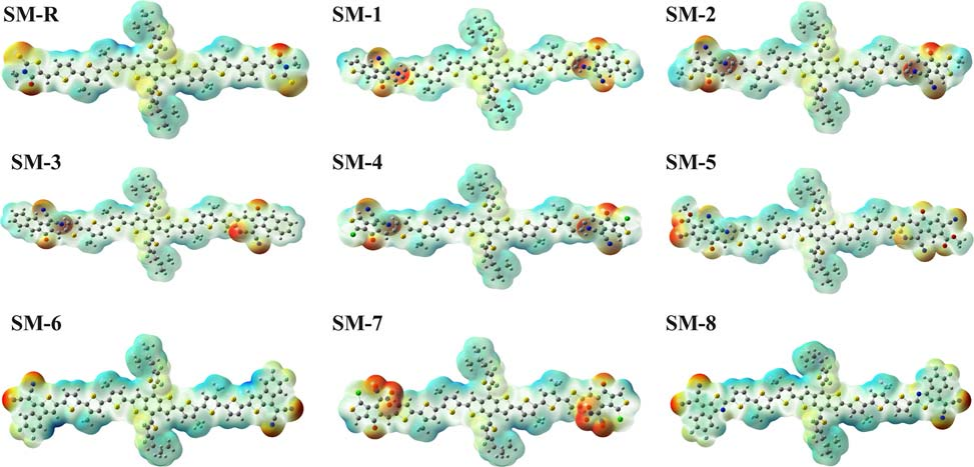
Molecular electrostatic potential (ESP) maps of the reference and designed donor molecules. Red regions represent electron rich zones, while blue areas denote electron deficient regions.

Molecular electrostatic potential (MEP) maps illustrate the spatial distribution of electron density across each donor framework, as shown in Fig. 3. The maps are colour coded such that red denotes electron rich (negative) regions, blue represents electron deficient (positive) regions, and green indicates nearly neutral potential.^51^ All designed donor molecules exhibit a clear polarity gradient extending from the electron rich fluorinated or sulfur containing donor core toward the terminal electron withdrawing acceptor units. The donor core and π-bridges appear moderately positive to near neutral, whereas the terminal groups carry strong negative potential, highlighting efficient ICT.

In the case of reference molecule SM-R, the negative potential localized around thiazolidinone carbonyl sites is significantly less pronounced, suggesting weaker electron withdrawing capability and hence relatively reduced internal electrostatic field throughout the molecule. On the other hand, SM-1 to SM-3 have more negative potentials at both molecular ends and thus show higher electron affinity and a stronger electrostatic gradient. SM-4 exhibits a broad region of negative potential around the cyano and carbonyl groups, while a partial positive area close to the C–Cl bond is due to halogen bonding, which can promote more ordered molecular packing. SM-5, having ester groups, also shows deep red regions at terminal sites consistent with LUMO stabilization. Among the series, the strongest internal polarization is observed in SM-6 and SM-8 with highly negative terminal zones and relatively positive central regions. SM-7 has significant terminal negativity though less uniform potentials owing to more twisted backbone geometry. From a device perspective, donor molecules with the strongly negative ends and a positive centre favour direct electron flow into acceptor domains for charge separation and reduce recombination simultaneously.^52,53^ Such polarity enhances exciton dissociation at D–A interfaces and strengthens ICT pathways.^54^ Therefore, SM-6 and SM-8 are the most promising candidates for achieving efficient charge separation and high electron mobility. SM-1 to SM-5 show moderate polarity enhancement, beneficial for improving absorption intensity and ICT while retaining a favourable *V*_oc_. SM-7 could assist charge dissociation but may require optimized donor pairing and morphological control to offset its structural twisting.

### Reduced density gradient analysis

Reduced density gradient (RDG) analysis offers an insight into weak non-covalent interactions that determine stability and conformation of the molecule in the ground state. RDG values are plotted against sign(*λ*_2_)ρ, where blue regions indicate attractive interactions (negative sign(*λ*_2_)ρ), green denotes van der Waals (vdW) contacts near zero, and red corresponds to steric repulsion (positive values).^55^ The RDG scatter plots and isosurfaces for the entire series are illustrated in Fig. S3. In all the donor molecules, a green band is continuous, indicating a significant extent of vdW stabilization along the π-conjugated backbone. The main differences are therefore observed in the blue and red zones. The presence of dense blue regions combined with narrow red regions implies the occurrence of weak attractive contacts and reduced steric repulsion. Broad red ones correspond to enhanced steric congestion at A–π–D–π–A junctions in which larger torsion occurs. The reference SM-R exhibits moderate green intensity, limited blue features and a notable red domain, establishing a baseline for steric load consistent with its nearly planar configuration. SM-1, SM-2, and SM-3 show a more significant blue population compared to the case of SM-R due to new attractive contacts induced by dicyanovinyl and carbonyl groups. Their broader red regions correspond to the larger dihedral angles listed in Table 1. SM-4 shows an extra blue peak at negative sign(*λ*_2_)ρ values, suggesting halogen assisted contacts involving chlorine. SM-5 strengthens the blue-green domain with only a moderate red contribution, implying enhanced stabilization without excessive crowding.

The best RDG properties are obtained for SM-6 and SM-8, which have the highest blue intensity and some of the narrowest red regions. These patterns imply multiple weak attractive interactions, such as S⋯F, F⋯H, and dipolar contacts, along with limited steric repulsion, consistent with their nearly planar backbones. SM-7 exhibits stronger blue density coupled with a wider red region, indicating the steric effects of nitro termini and the resulting backbone twist. Planar geometry, near π–π stacking, and less energetic disorder of the blue-green RDG signatures lead to higher carrier mobility and charge extraction efficiency. Areas dominated by red, in turn, show the structural twisting and discouragement of packing due to reduced delocalization.^56^ According to these results, SM-6 and SM-8 are identified as the optimal donors due to better charge separation and transport. SM-5 is also characterized by good stabilization, while SM-1 to SM-4 show beneficial interaction gains, although with a certain torsional penalty.

### Density of states analysis

The spatial distribution of frontier orbitals shows how terminal group substitution governs electronic coupling and ICT directionality in the designed donor molecules.^57^ Density of states (DOS) and projected DOS (PDOS) calculations were performed at the B3LYP level with the 6-31G(d,p) basis set. The HOMO remains delocalized along the conjugated backbone. The bridge carries between 57.0% and 62.5% of the HOMO across the series, while the donor core contributes between 26.5% and 33.8%. This backbone centered HOMO supports continuous hole transport and stabilizes the ionization potential. In contrast, the LUMO progressively migrates toward the terminal acceptors as electron withdrawing strength increases, rising from 37.1% acceptor character in SM-R to 84.2% in SM-6 and 83.8% in SM-8. This evolution establishes a clear donor-to-acceptor polarization that enhances electron affinity and narrows the *E*_g_.^57^ Molecules with moderate LUMO localization, including SM-1 at 55.7%, SM-2 at 57.2%, and SM-3 at 57.8%, display balanced ICT, red shifted absorption, and limited voltage loss. Halogen substitution in SM-4 preserves a similar LUMO composition at 57.2% and can promote directional halogen π interactions that support molecular ordering without disturbing energetic alignment. Strengthening the termini in SM-5 at 61.9%, SM-6 at 84.2%, SM-7 at 65.1%, and SM-8 at 83.8% further confines the LUMO to the molecular periphery, producing measurable *E*_g_ contraction and reduced e–h coupling. SM-6 and SM-8 combine the strongest peripheral LUMO localization with near planar backbones, a configuration that favours strong ICT character, extended light harvesting, and efficient exciton dissociation.

**Fig. 4:**
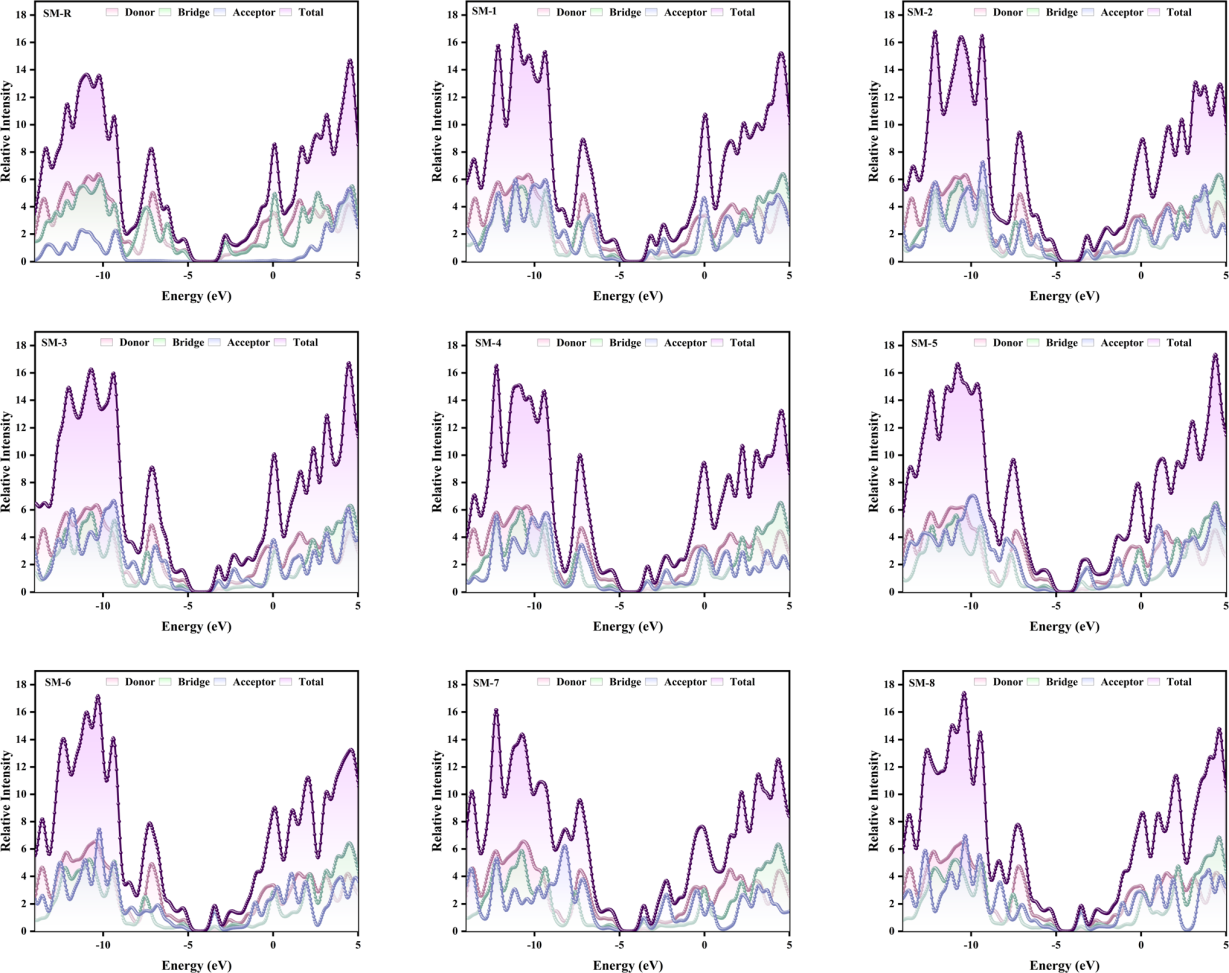
Density of states (DOS) and projected DOS (PDOS) of the reference and designed donor molecules.

Donor core participation in the LUMO decreases from 11.9% in SM-R to 1.0% in SM-6 and 1.0% in SM-8, confirming spatial decoupling of electron and hole densities. The bridge retains the dominant HOMO share, for example 62.5% in SM-R, 60.6% in SM-1, 60.3% in SM-2, 60.2% in SM-3, 58.9% in SM-4, 57.1% in SM-5, 61.7% in SM-6, 57.0% in SM-7, and 61.0% in SM-8. This electronic conduit links donor and acceptor segments and translates terminal group stabilization into observable *E*_g_ narrowing and lower internal reorganization energy. Terminal group engineering controls the spatial origin of the frontier orbitals and thereby tunes ICT strength, absorption bandwidth, and interfacial energy offsets. SM-6 and SM-8 are predicted to deliver strong light harvesting and efficient exciton dissociation. SM-1 to SM-4 maintain balanced alignment that supports voltage preservation. SM-5 and SM-7 occupy an intermediate regime where performance will depend on donor pairing and film morphology. The electronic trends discussed above are substantiated by the DOS data in Fig. 4 and Table S1, which quantitatively trace the evolution of orbital localization across the molecular series.

### Molecular polarity and solution processability

The dipole moment (*D*) plays an important role in linking a molecule's electronic structure with how well it dissolves and performs in devices. Molecules with higher dipole moments interact more strongly with solvents, which helps them dissolve better and mix more easily with common casting solvents.^58^ Higher molecular polarity in D–A blends enhances material compatibility and promotes the formation of organized phase domains. A pronounced D induces an internal electric field that modulates local energy levels and generates favourable interfacial dipoles at D–A junctions. These effects improve energy level alignment, facilitate charge transfer, and lower the *E*_b_ through enhanced dielectric screening. As a result, exciton separation becomes more effective and charge recombination is minimized, which ultimately enhances the overall device performance. In this work, computed gas phase and solvent phase dipole moments are used to evaluate how different terminal acceptor substitutions modulate polarity. The reference molecule SM-R exhibits the smallest dipole moment in both phases. In the gas phase, D ranges from 2.23 *D* for SM-R to 9.27 *D* for SM-8. The order is SM-8 > SM-5 > SM-1 > SM-2 > SM-6 > SM-3 > SM-7 > SM-4 > SM-R. In chloroform, the range extends from 2.76 *D* for SM-R to 11.68 *D* for SM-7, following the order SM-7 > SM-5 > SM-6 > SM-2 > SM-8 > SM-1 > SM-3 > SM-4 > SM-R. SM-7 and SM-6 show increase in dipole moment in the solvent phase, suggesting that the polarizable medium stabilizes the more polarized ground state associated with the stronger acceptors. All computed values are summarized in Table S2. These polarity variations influence key device related processes. A larger D minimizes e–h overlap, facilitates exciton dissociation at D–A interfaces, and improves solute–solvent interactions during film formation, thereby enhancing D–A miscibility.^59^ Conversely, excessively high dipoles may induce morphological disorder and lower *V*_oc_. Lower dipoles reduce this risk but may limit the driving force for separation and solubility enhancement.^60^

Among the designed molecules, SM-R is least polar as expected based on its weak ICT characteristics and poor processability. SM-1 and SM-2 possess the moderate dipole moments in both phases, which indicate good solubility and can favour interfacial charge separation as well as maintain a reasonable *V*_oc_. SM-3 shows an intermediate dipole in solvent, lower than SM-1 and SM-2 but higher than SM-R. This level of polarity should moderately enhance solubility and interfacial polarization. SM-4 remains weakly polar in both phases, indicating that its film quality will rely more on molecular packing. SM-5 exhibits high solubility as well as a large dipole moment in the solvent phase. SM-6, having a high solvent phase dipole in combination with a near planar backbone, also has anticipated excellent processability, efficient exciton dissociation and coherent charge transport. SM-7 attains the highest dipole in solvent and is promising for separation and miscibility. In gas and solvent phases, SM-8 is the most polar molecule. It manifests a good charge separation and transportation once the energy level alignment at the interface is well established. This analysis shows that molecular polarity can be tuned by changing the terminal acceptor groups and used to optimize solubility, interface properties and film morphology for superior OSC device performance.

### Absorption maxima and solvent dependent spectral shifts

**Fig. 5:**
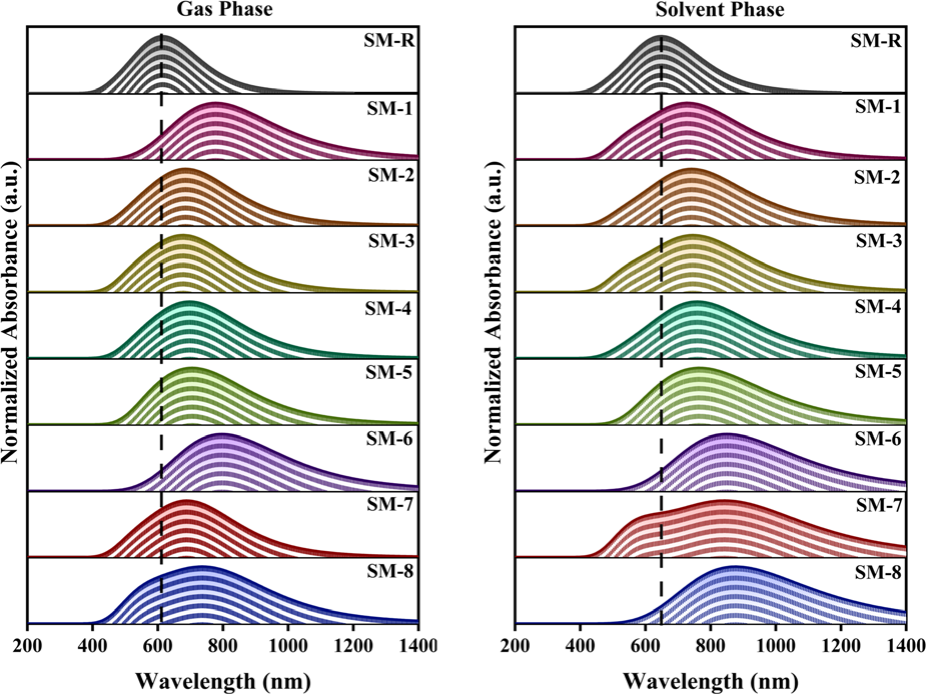
UV-vis spectra of SM-R and designed molecules in gas and solvent phases.

Both in the gas and solvent phase, the absorption spectra (*λ*_max_) of all designed molecules show a red shift in their *λ*_max_ values compared to the reference molecule SM-R. As shown in Fig. 5, SM-R displays a *λ*_max_ of 629 nm in the gas phase, indicating its wider *E*_g_ and weaker ICT behaviour. All designed molecules show red shifted *λ*_max_, confirming enhanced π-electron delocalization, as summarized in Table S3. Among designed donor molecules, SM-6 and SM-1 display the most red-shifted spectra. Red shifts in SM-2, SM-3, and SM-4 show *E*_g_ reduction due to terminal substitution, confirming effective modulation of electronic coupling through acceptor side engineering. In the solvent phase, all designed donor molecules show further red shifted absorption compared with SM-R, confirming solvent induced stabilization of the excited states, as shown in Table S4. The extended *λ*_max_ is observed for SM-8 at 903 nm, followed by SM-7 at 898 nm and SM-6 at 874 nm. These extended red shifts provide evidence for enhanced ICT nature and high photon absorption. However, the red shift observed in SM-8 may be related to a deeper LUMO level and a smaller *E*_g_. This can result in a lower *V*_oc_ and higher non-radiative recombination losses.^61^ While extending absorption into the NIR enhances light harvesting, excessive *E*_g_ narrowing can introduce important trade-offs. Very small *E*_g_ may reduce the achievable *V*_oc_, increase non-radiative recombination losses, and weaken the energetic driving force for exciton dissociation at D–A interfaces. In addition, strong D–A interactions associated with narrow *E*_g_ systems can promote excessive aggregation, potentially leading to unfavourable morphology and increased recombination in practical devices. Molecules with moderate red shifts, such as SM-1 and SM-4 are expected to achieve a better balance providing broad optical coverage while maintaining favourable voltage and morphological stability. The shifts in *λ*_max_ demonstrate successful control of energy levels through end-group engineering. Among the series, SM-6 and SM-8 show the strong light harvesting potential, while SM-1 and SM-4 combine extended absorption with improved voltage retention, making them promising donor candidates for high performance OSCs.

### Excitation energy and oscillator strength

The excitation energy (*E*_*x*_) represents the vertical energy difference between the ground state (S_0_) and the lowest excited state (S_1_) obtained from TD-DFT calculations. A smaller *E*_*x*_ corresponds to a longer wavelength *λ*_max_, enabling broader absorption of the solar spectrum and more efficient photon harvesting, which contributes to higher photocurrent generation.^61^ Across both gas and solvent phases, all designed donor molecules exhibit lower *E*_*x*_ values than the reference SM-R, confirming their narrower *E*_g_ and stronger ICT character. In the gas phase, the smallest *E*_*x*_ values are obtained for SM-6 and SM-1, followed by SM-8 and SM-5. In the solvent phase, the ordering changes slightly, with SM-8 and SM-7 showing the lowest *E*_*x*_ values, and SM-6 and SM-5 following closely. This solvent dependence reflects stabilization of the more polar S_1_ states in the designed series, consistent with the ICT behaviour identified from the FMO analysis. The computed *E*_*x*_ values are summarized in Table S3 for the gas phase and in Table S4 for the solvent phase. Oscillator strengths (*f*) remain relatively high across the series, indicating that the lowest energy transitions maintain strong intensity even with substantial ICT contributions. Most molecules display slightly larger *f* values in chloroform than in the gas phase, consistent with an enhanced transition dipole in a polarizable medium. Two exceptions are observed: SM-8 shows very low *E*_*x*_ and pronounced red shifts, while SM-7 shows a similar but weaker effect, along with a modest reduction in *f* in solvent compared with the gas phase. In contrast, SM-1 achieves a considerable red shift while retaining one of the highest *f* values in the solvent, which is advantageous for light harvesting performance. The complete *f* values are listed in Table S3 for gas and Table 4 for the solvent phase.

### Light harvesting efficiency and excited state lifetime

Light harvesting efficiency (LHE) quantifies the ability of a molecule to absorb incident photons at a given transition. It is evaluated from the *f via* the following equation:

1 LHE = 1 − 10^−*f*^

where larger *f* yields higher LHE, bounded between 0 and 1. A high LHE increases the fraction of absorbed photons within the active layer and consequently enhances the *J*_sc_ at realistic film thicknesses. High LHE at longer wavelengths is particularly beneficial, as it improves overlap with the solar spectrum.^62^ The LHE values derived from TD-DFT calculations are summarized in Table S5. All designed molecules exhibit large *f* for the lowest energy ICT transition in both gas and solvent phases, resulting in high LHE across the series. Molecules that combine a red shifted absorption band with sustained *f* deliver the most desirable optical response. SM-1 shows the highest *f* value in the solvent and thus the largest LHE, whereas SM-6 and SM-8 extend absorption to longer wavelengths with only modest reductions in *f*, preserving efficient photon capture in the visible near IR region.

The radiative lifetime (*τ*) of the lowest singlet excited state was estimated from *E*_*x*_ and *f via* the following equation:

2 *τ* = 1.499/*fE*_*x*_^2^

where *E*_*x*_ is expressed in eV and *τ* in nanoseconds (ns). A longer *τ* increases the temporal window for excitons to reach D–A interfaces before recombination, thereby improving charge generation probability.^63^ As summarized in Table S5, the sequence of *τ* follows the order: SM-8 > SM-6 > SM-7 > SM-5 > SM-4 > SM-2 > SM-3 > SM-1 > SM-R, corresponding to *τ* = 0.420, 0.369, 0.324, 0.260, 0.191, 0.176, 0.173, 0.165, and 0.148 ns, respectively. End group tuning lowers *E*_*x*_ for all designed molecules relative to SM-R, resulting in consistently longer lifetimes. The largest *τ* values occur for SM-8 and SM-6, where the excitation energy is smallest and the *f* remains moderate, indicating strong ICT character with minimal quenching of transition intensity. Molecules that exhibit an extreme red shift accompanied by diminished *f* show weaker lifetime enhancement because the *f* term counter balances the effect of small *E*_*x*_. Conversely, SM-1 retains a high *f* at a slightly shorter wavelength, producing very large LHE but a moderate *τ*. This trend demonstrates that a controlled reduction in *E*_*x*_, while maintaining moderate *f*, effectively extends *τ*, facilitating exciton migration and charge separation. SM-6 and SM-8 therefore emerge as the most promising candidates for enhanced LHE and prolonged *τ*, provided that suitable donor materials sustain interfacial energy offsets and stable blend morphology.

### Transition density matrix analysis

**Fig. 6:**
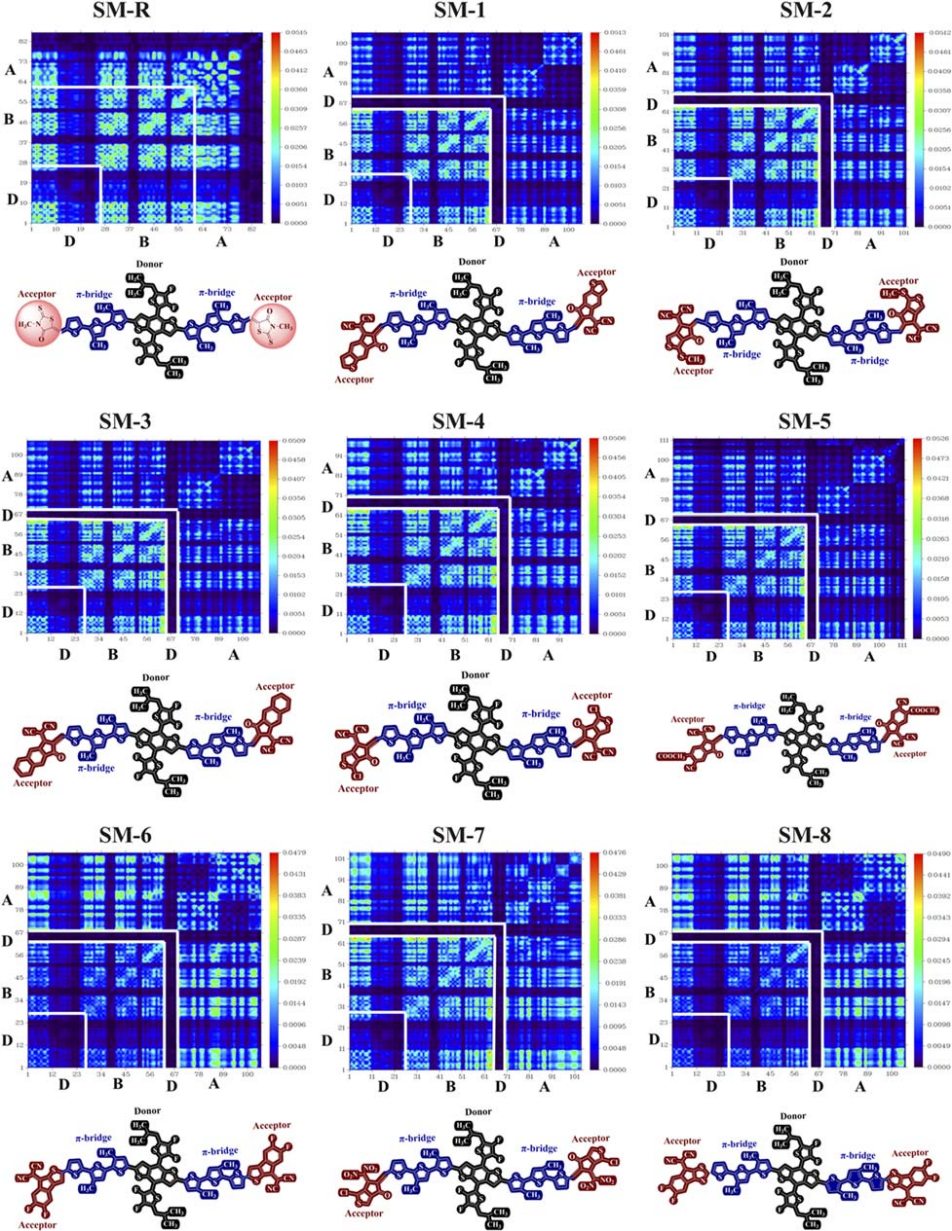
Transition density matrix maps of the reference and designed donor molecules. The diagonal regions represent local excitations, while off-diagonal elements indicate charge transfer between molecular fragments.

The transition density matrix (TDM) provides a spatial picture of electron redistribution during the S_0_ → S_1_ excitation, identifying regions where electron density is depleted (hole) and where it accumulates (electron). After dividing each molecule into donor, bridge, and acceptor segments, diagonal elements represent local excitations confined within one fragment, whereas off-diagonal elements correspond to ICT between fragments. Intense donor-to-acceptor or bridge-to-acceptor features thus signify strong ICT, while dominant donor-to-donor or bridge-to-bridge signals indicate a more localized excitation.^64^ A larger off-diagonal electron–hole coupling therefore implies increased spatial separation between the excited electron and hole, characteristic of CT dominated excited states. Such delocalized excited states favour intermolecular electronic overlap and ordered π–π stacking by reducing exciton localization. This behaviour is beneficial for efficient charge transport, as it facilitates charge delocalization and intermolecular hopping in the solid state. In Fig. 6, the reference molecule SM-R shows strong diagonal intensity over the donor and bridge regions, with only weak population on the acceptor segment.

This pattern reflects a predominantly local excitation, consistent with its larger *E*_g_ and shorter *λ*_max_. Introducing dicyanovinyl and carbonyl end groups in SM-1, SM-2, and SM-3 increases the off-diagonal coupling between the donor, bridge and acceptor domains. The hole remains localized on the donor and bridge, while the electron density relocates toward the acceptor termini, confirming enhanced ICT and explaining the observed reduction in *E*_g_ and red-shift in *λ*_max_. SM-4 retains strong bridge-to-acceptor transfer with a small residual local component, in line with its moderate spectral shift and the ordering influence of halogen substitution. SM-5 exhibits even greater acceptor population with effective mediation through the bridge, indicating reduced e–h overlap and improved exciton separation.

The most transfer dominated maps are obtained for SM-6 and SM-8. Their off-diagonal backbone-to-acceptor blocks are the most intense in the series, while diagonal features are weak. This pattern aligns with their acceptor localized LUMOs and near planar backbones, supporting efficient splitting of the photoexcited state and well-defined electron transport channels within acceptor rich domains. SM-7 also displays strong ICT character, although a noticeable local component persists on the backbone due to its larger torsion and slightly wider *E*_g_. From a device perspective, TDM maps dominated by donor-to-acceptor and bridge-to-acceptor transfer reduce e–h overlap, lower *E*_*x*_, and promote interfacial charge separation. Designs that combine strong transfer with structural planarity, particularly SM-6 and SM-8, are therefore expected to yield robust photocurrent generation and coherent electron transport within the acceptor phase. Molecules such as SM-1 to SM-4 provide a balanced compromise that preserves *V*_oc_ while enhancing light absorption and exciton dissociation.

### Exciton binding energy

The exciton binding energy (*E*_b_) is the energy of coulombic attraction between a photoexcited electron and hole. It is an important factor of the charge separation efficiency in OSCs. A smaller *E*_b_ is more beneficial for exciton dissociation into free carriers in an internal electric field, consequently promoting the charge generation and photocurrent.^65^ In this study, *E*_b_ was obtained from TD-DFT calculations in both the gas and solvent phases. The computed values are summarized in Table 3. In the gas phase, the *E*_b_ of reference molecule SM-R is 0.26 eV. Most of the designed donors show reduced *E*_b_, indicating weaker e–h coulombic attraction and more efficient charge separation. SM-3, SM-4, SM-5, and SM-6 display nearly similar values, indicating efficient exciton delocalization and desirable charge transfer contribution. The minimum obtained *E*_b_ is 0.04 eV for SM-7. This indicates almost zero barrier separation at the interface, but its large polarized structure may increase the non-radiative recombination losses. In comparison, SM-1 has a high *E*_b_ of 0.46 eV, which implies more intense e–h coupling in this molecule and less exciton separation compared to other designed donor molecules. In the solvent phase, all designed donors show higher *E*_b_ values. The lowest *E*_b_ is seen for SM-6 with 0.30, signifying its better separation ability of e–h pairs.

**Table 3:** Calculated exciton binding energies of the reference and designed donor molecules in gas and solvent phases

Molecules	*E*_b_ (eV) gaseous	*E*_b_ (eV) solvent
SM-R	0.26	0.36
SM-1	0.46	0.36
SM-2	0.23	0.35
SM-3	0.21	0.35
SM-4	0.22	0.34
SM-5	0.22	0.36
SM-6	0.22	0.30
SM-7	0.04	0.37
SM-8	0.14	0.32

The pronounced red shift in absorption observed upon substitution with strongly electron withdrawing terminal acceptors originates from enhanced ICT and preferential stabilization of the LUMO relative to the HOMO, leading to a reduced *E*_g_. The increased D–A interaction promotes electronic delocalization along the molecular backbone, thereby lowering the *E*_*x*_ and extending absorption into the NIR. In parallel, the reduced *E*_b_ indicate weakened coulombic attraction between photogenerated electron–hole pairs, arising from increased charge delocalization and enhanced dielectric screening. Lower *E*_b_ facilitates exciton dissociation at D–A interfaces, which is important for efficient charge generation and improved photocurrent in OSCs. From the perspective of exciton physics in organic semiconductors, such a reduction in *E*_b_ is particularly important, as the low dielectric constant of organic materials typically leads to strongly bound excitons and inefficient charge generation. Lower *E*_b_ facilitates exciton dissociation at D–A interfaces, thereby addressing a key limitation in OSCs related to inefficient charge separation and non-radiative recombination. These results indicate that donor molecules with planar backbones and balanced polarity, particularly SM-6, provide an effective balance between *E*_b_ and charge separation efficiency, making them strong candidates for high performance OSCs.

### Reorganization energy

Reorganization energy (*λ*) is a key factor in determining the photovoltaic performance of materials designed for use in OSCs. It provides a measure of the energy associated with the geometric relaxation of a molecule and its surrounding environment upon a change in its electronic state, such as during the excitation and charge transfer processes.^66^ A fundamental purpose of calculating *λ* is to understand the influence of molecular reorganization on the charge transfer properties, particularly electron and hole mobility within the active layer of the device. The *λ* can be decomposed into two constituent components: the internal reorganization energy (*λ*_int_) and the external reorganization energy (*λ*_ext_). The term *λ*_int_ arises from changes in the internal vibrational geometries of the molecule itself, while the term *λ*_ext_ originates from the polarization response of the surrounding medium. The value of *λ*_int_ was obtained with the four-point Marcus scheme, as it is influenced by the energy differences between the anions, cations, and neutral species at their optimized and distorted geometries. Calculations were carried out at the B3LYP/6-31G d,p level, and the electron and hole components were determined from the Marcus relations described in the SI. The computed values are summarized in Table 4. The calculated *λ*_e_ and *λ*_h_ values provide key insight into the charge transfer characteristics of the designed donor molecules. A consistent trend emerges in which most of the new donors, from SM-1 to SM-6 and SM-8, show a marked reduction in *λ*_e_ compared with the reference molecule SM-R, which has a value of 0.178 eV.

**Table 4:** Calculated electron and hole reorganization energies of the reference and designed donor molecules

Molecules	*λ*_e_ (eV)	*λ*_h_ (eV)
SM-R	0.177777	0.195633
SM-1	0.141741	0.214531
SM-2	0.131139	0.211266
SM-3	0.121907	0.209209
SM-4	0.118862	0.215571
SM-5	0.110845	0.216561
SM-6	0.091052	0.182664
SM-7	0.209952	0.251596
SM-8	0.092494	0.177078

The designed donors exhibit *λ*_e_ values ranging from 0.141 to 0.091 eV, indicating enhanced structural rigidity upon reduction and improved interfacial CT. Low *λ*_e_ reduces the energetic penalty associated with electron hopping, thereby facilitating faster and more efficient electron transport. This is particularly important for suppressing charge accumulation and recombination at D–A interfaces, ultimately contributing to improved charge extraction and device performance in OSCs. In contrast, *λ*_h_ values remain relatively high and vary less systematically, with most exceeding the SM-R reference value of 0.196 eV. SM-7 deviates from this overall pattern, showing the largest *λ* for both charge carriers, with *λ*_e_ of 0.210 eV and *λ*_h_ of 0.252 eV, reflecting pronounced molecular relaxation in both charged states and reduced charge transfer efficiency. Among the series, SM-6 with *λ*_e_ of 0.091 eV and *λ*_h_ of 0.183 eV, and SM-8 with *λ*_e_ of 0.092 eV and *λ*_h_ of 0.177 eV, emerge as the most promising donor materials. Their combination of very low *λ*_e_ and moderately small *λ*_h_ suggests efficient and selective hole transport together with stable charge separation at the D–A interface. This balance of rigidity and mobility is essential for achieving high photocurrent generation and superior power conversion efficiency in OSCs based on D–A systems.

### Device level photovoltaic parameters

#### Open circuit voltage

Open circuit voltage (*V*_oc_) defines the maximum potential difference that a photovoltaic device can deliver under illumination in the absence of an external current. In OSCs, *V*_oc_ is primarily governed by the energy level alignment between the HOMO of the donor and the LUMO of the acceptor, which determines the thermodynamic driving force for charge separation. A deeper HOMO in the donor or a lower LUMO in the acceptor increases this voltage gap, leading to higher *V*_oc_. But, the actual value is lower than the theoretical maximum due to non-radiative recombination, interfacial energy losses, and exciton binding effects. Optimizing *V*_oc_ therefore requires minimizing energetic losses while preserving efficient charge transfer across the D–A interface. In the designed donor molecules, the *V*_oc_ is directly affected by tuning the HOMO level through structural modification. This shows a clear connection between molecular design and device performance. The *V*_oc_ of all designed donor molecules was calculated using the Scharber relation:3
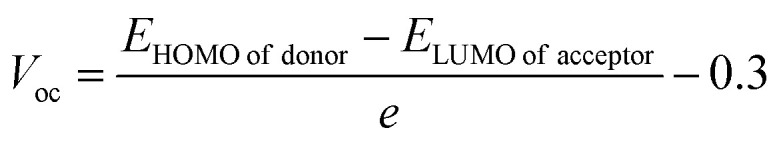


A 0.3 eV offset was included to account for energy losses caused by exciton dissociation and non-radiative recombination. The fixed energy offset of 0.3 eV is adopted here as a conventional approximation to enable comparative evaluation across the donor series; however, it is recognized that optimized non-fullerene systems may exhibit lower non-radiative energy losses in experimentally realized devices.

The reference acceptor in this study is the fullerene derivative PC_61_BM, which has HOMO and LUMO energy levels of −6.10 eV and −3.70 eV, respectively. The calculated *V*_oc_ for SM-R and the designed donor molecules compared with PC_61_BM are listed in Table 5. The computed *V*_oc_ values follow the sequence: SM-5 > SM-7 > SM-4 > SM-8 > SM-1 = SM-3 > SM-2 = SM-6 > SM-R. This trend demonstrates that all the designed donor molecules exhibit higher *V*_oc_ values than the reference SM-R, primarily due to their deeper HOMO energy levels, which improve energetic alignment with the LUMO of PC_61_BM. The higher *V*_oc_ values, particularly for SM-A5 and SM-A7, suggest a reduced recombination probability and a stronger thermodynamic driving force for charge separation. These results suggest that the newly developed donors, when paired with PC_61_BM, possess favourable energy level alignment for efficient electron extraction and high *V*_oc_, confirming their potential as promising donor materials for next generation OSCs.

**Table 5:** Calculated photovoltaic parameters of the reference and designed donor molecules

Molecules	*V*_oc_ (eV)	Normalized *V*_oc_	FF %	*E*_loss_ (eV)
SM-R	1.09	42.12	0.8897	1.14
SM-1	1.17	45.22	0.8956	0.82
SM-2	1.16	44.83	0.8949	0.80
SM-3	1.17	45.22	0.8956	0.78
SM-4	1.28	49.47	0.9026	0.63
SM-5	1.39	53.72	0.9087	0.46
SM-6	1.16	44.83	0.8949	0.55
SM-7	1.33	51.40	0.9055	0.42
SM-8	1.22	47.15	0.8989	0.47

#### Fill factor

The fill factor (FF) is a key determinant of the PCE of an OSC. It was estimated using the semi-empirical relation given by Scharber and co-workers:4
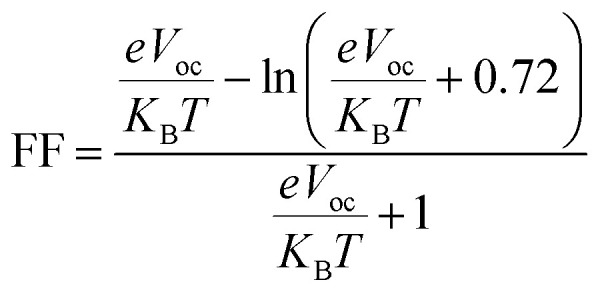
where *e* is the elementary charge, *k*_B_ is the Boltzmann constant, *T* is the temperature (taken as 300 K). The corresponding FF values are summarized in Table 5. The FF follows the same trend as the *V*_oc_, indicating that improved *E*_g_ and *V*_oc_ in the designed donors enhances FF. The FF values in the theoretical calculations predict a great improvement of the overall photovoltaic performance of these novel donor molecules. SM-4, SM-5 and SM-7 exhibit the highest FF values. The high FF values in SM-4, SM-5 and SM-7 indicate that they are possibly able to provide high PCEs, and their applicability in the generation of high performance donor materials for next generation OSCs.

#### Energy loss

The energy loss (*E*_loss_) is an important factor in controlling the total efficiency of OSCs, because it dominates the proportion of absorbed solar power that can be utilized to generate electrical output. Higher *E*_loss_ leads to smaller *V*_oc_ and lower PCE, whereas the minimization of *E*_loss_ improves the output and stability of devices.^67^ The overall *E* loss for each donor molecule was approximated using the following relationship:

5 *E*_loss_ = *E*_g_ − *eV*_oc_

The reference molecule SM-R shows an *E*_loss_ of 1.14 eV, while all the designed donor molecules display much smaller values. SM-2, SM-3, and SM-4 show moderate reductions with energy losses of 0.80, 0.78, and 0.63 eV, respectively. Further improvement is seen for SM-5, SM-6, and SM-8, which show lower values of 0.46, 0.55, and 0.47 eV. The smallest energy loss of 0.42 eV for SM-7 indicates minimal voltage loss and more efficient energy conversion, making it the most favourable donor in the series. The computed *E*_loss_ values for all molecules are summarized in Table 5. The lower *E*_loss_ found in all the designed donor molecules compared to SM-R shows that their frontier orbital alignment is better and non-radiative recombination is reduced. SM-4, SM-5 and SM-7 stand out because of their small *E*_loss_, high *V*_oc_, and large FF. These features suggest that these donors can support stronger photovoltaic performance and are good candidates for efficient OSCs. The reported device level parameters are theoretical estimates and may not fully translate to fabricated devices due to morphology dependent effects such as phase separation, molecular packing, trap formation, and interfacial recombination. In practice, non-ideal D–A miscibility, energetic disorder, and contact related losses can reduce charge extraction and increase recombination, thereby lowering the measured *V*_oc_, FF, and PCE. Therefore, experimental device performance will depend strongly on blend morphology and processing conditions in addition to intrinsic molecular properties.

## Conclusion

The study has elucidated that the terminal group engineering of benzodithiophene-based donor molecules is an effective strategy to modulate their electronic properties and photovoltaic performance. Replacing the terminal acceptors with stronger electron withdrawing acceptors enhances orbital interactions, promotes ICT and red shifts optical absorption toward the NIR. These modifications enhance the charge separation process and enable more effective utilization of the solar spectrum. Among the designed molecules, SM-6 and SM-8 exhibit low *λ*_e_ of 0.091 eV and 0.092 eV and low *λ*_h_ of 0.183 eV and 0.177 eV. These values indicate reduced *λ*_int_ for hole transport in the donor phase and efficient electron transfer at the D–A interface. In addition, their *τ* values of 0.369 ns and 0.420 ns suggest favourable exciton diffusion and effective charge extraction. At the device level, SM-5, SM-7, and SM-4 deliver high *V*_oc_ of 1.39 eV, 1.33 eV, and 1.28 eV with FF exceeding 0.90. Notably, the smallest *E*_loss_ of 0.42 eV observed for SM-7 reflects suppressed non-radiative recombination. The identified donor molecules, particularly SM-6, SM-7, and SM-8, therefore represent promising candidates for future experimental synthesis and device fabrication based on the design principles established in this study. These findings demonstrate that terminal group engineering is a powerful and rational design approach for achieving high performance and low energy loss donor materials for next generation OSCs.

## Author contributions

S. M. K. A. Naqvi and R. A. Khera conceived and designed the study; S. Khanam, Z. F. Khudair, I. H. Jaghdam, and R. F. Mehmood carried out the computational modeling and figure preparation; M. Imran and M. S. Soliman contributed to data analysis and interpretation; A. M. Shawky, R. Bousbih and S. Khanam participated in writing and editing the manuscript.

## Conflicts of interest

The authors declare that they have no known competing financial interests or personal relationships that could have appeared to influence the work reported in this paper.

## Supplementary Material

NA-008-D5NA01002K-s001

NA-008-D5NA01002K-s002

NA-008-D5NA01002K-s003

NA-008-D5NA01002K-s004

NA-008-D5NA01002K-s005

## Data Availability

All data provided and/or analysed during this study were included as figures and tables in this article and its supplementary information (SI). Supplementary information is available. See DOI: https://doi.org/10.1039/d5na01002k.

## References

[cit1] Zhang R., Chen H., Wang T., Kobera L., He L., Huang Y., Ding J., Zhang B., Khasbaatar A., Nanayakkara S., Zheng J., Chen W., Diao Y., Abbrent S., Brus J., Coffey A. H., Zhu C., Liu H., Lu X., Jiang Q., Coropceanu V., Brédas J.-L., Li Y., Li Y., Gao F. (2025). Nat. Energy.

[cit2] Li C., Song J., Lai H., Zhang H., Zhou R., Xu J., Huang H., Liu L., Gao J., Li Y., Jee M. H., Zheng Z., Liu S., Yan J., Chen X.-K., Tang Z., Zhang C., Woo H. Y., He F., Gao F., Yan H., Sun Y. (2025). Nat. Mater..

[cit3] Chen L., Zhang Y., Chen Z., Dong Y., Jiang Y., Hua J., Liu Y., Osman A. I., Farghali M., Huang L., Rooney D. W., Yap P.-S. (2024). Environ. Chem. Lett..

[cit4] Forberich K., Troisi A., Liu C., Wagner M., Brabec C. J., Egelhaaf H.-J. (2024). Adv. Funct. Mater..

[cit5] Wu Z., Shi B., Yu J., Sha M., Sun J., Jiang D., Liu X., Wu W., Tan Y., Li H., Huang S., Wang J., Liu J., Zhang C., Ma X., Cui L., Ye L., Zhang F., Cao B., Chen Y., Ji Z., Chen F., Hao X., Li G., Yin H. (2024). Energy Environ. Sci..

[cit6] Zhu J., Xia J., Li Y., Li Y. (2025). ACS Appl. Mater. Interfaces.

[cit7] Zhang Y., Xia H., Yu J., Yang Y., Li G. (2025). Adv. Mater..

[cit8] Abubaker S. A., Pakhuruddin M. Z. (2024). Renewable Sustainable Energy Rev..

[cit9] Jungbluth A., Cho E., Privitera A., Yallum K. M., Kaienburg P., Lauritzen A. E., Derrien T., V Kesava S., Habib I., Pratik S. M., Banerji N., Brédas J.-L., Coropceanu V., Riede M. (2024). Nat. Commun..

[cit10] Lee J.-W., Park J. S., Jeon H., Lee S., Jeong D., Lee C., Kim Y.-H., Kim B. J. (2024). Chem. Soc. Rev..

[cit11] Ahmad N., Yuan J., Zou Y. (2025). Energy Environ. Sci..

[cit12] Han J.-H., Zhao Z.-W., Pan Q.-Q., Wang L.-L., Zhou H.-P., Su Z.-M. (2025). ACS Appl. Mater. Interfaces.

[cit13] Yao H., Cui Y., Qian D., Ponseca C. S. J., Honarfar A., Xu Y., Xin J., Chen Z., Hong L., Gao B., Yu R., Zu Y., Ma W., Chabera P., Pullerits T., Yartsev A., Gao F., Hou J. (2019). J. Am. Chem. Soc..

[cit14] Hu D., Tang H., Chen C., Huang P., Shen Z., Li H., Liu H., Petoukhoff C. E., Jurado J. P., Luo Y., Xia H., Fong P. W. K., Fu J., Zhao L., Yan C., Chen Y., Cheng P., Lu X., Li G., Laquai F., Xiao Z. (2024). Adv. Mater..

[cit15] Guo L., Wu L., Jia T., Zhang H., Song J., Xie X., Jee M. H., Ma H., Liu S., Lu G., Woo H. Y., Wang Z., Gao F., Sun Y. (2025). Angew. Chem., Int. Ed..

[cit16] Fu J., Yang Q., Huang P., Chung S., Cho K., Kan Z., Liu H., Lu X., Lang Y., Lai H., He F., Fong P. W. K., Lu S., Yang Y., Xiao Z., Li G. (2024). Nat. Commun..

[cit17] Zhou D., Wang Y., Yang S., Quan J., Deng J., Wang J., Li Y., Tong Y., Wang Q., Chen L. (2024). Small.

[cit18] Kazim Abbas Naqvi S. M., Abbas F., Bibi S., Shehzad M. K., Alhokbany N., Zhu Y., Long H., Vasiliev R. B., Nazir Z., Chang S. (2024). RSC Adv..

[cit19] Sanviti M., Marina S., Rodriguez-Martínez X., Asatryan J., Di Lisio V., Hultmark S., Gutierrez J., Solano E., Rech J. J., Solla E. L., You W., Tercjak A., Vázquez M. E., Cangialosi D., Müller C., Ade H., Martin J. (2025). Adv. Funct. Mater..

[cit20] Rodríguez-Martínez X., Marina S., Harillo-Baños A., Campoy-Quiles M., Martin J. (2025). J. Mater. Chem. C.

[cit21] Lin Y., Ma Z., Tang Z. (2025). Mater. Horiz..

[cit22] Pratik S. M., Kupgan G., Brédas J.-L., Coropceanu V. (2025). Energy Environ. Sci..

[cit23] Ma R., Liu T., Luo Z., Guo Q., Xiao Y., Chen Y., Li X., Luo S., Lu X., Zhang M., Li Y., Yan H. (2020). Sci. China Chem..

[cit24] Sağlamkaya E., Shadabroo M. S., Tokmoldin N., Melody T. M., Sun B., Alqahtani O., Patterson A., Collins B. A., Neher D., Shoaee S. (2024). Mater. Horiz..

[cit25] Gao W., Ma R., Dela Peña T. A., Yan C., Li H., Li M., Wu J., Cheng P., Zhong C., Wei Z., Jen A. K.-Y., Li G. (2024). Nat. Commun..

[cit26] Javed M., Akram W., Ali Z., Shahzad N., Shahid M., Chotana G. A., Khan J. I., Min J., Altaf M., Nielsen C. B., Ashraf R. S. (2025). J. Mater. Chem. C.

[cit27] Mahmood A., Hu J., Tang A., Chen F., Wang X., Zhou E. (2018). Dyes Pigments.

[cit28] Li M. Q., Jeong M., Park B., Alam S., Lee J., Lee H., Lee J., Cho K. (2025). Adv. Funct. Mater..

[cit29] Mahmood A., Hu J.-Y., Xiao B., Tang A., Wang X., Zhou E. (2018). J. Mater. Chem. A.

[cit30] Ge J., Chen Z., Ye Q., Xie L., Song W., Guo Y., Zhang J., Tong X., Zhang J., Zhou E., Wei Z., Ge Z. (2023). ACS Appl. Mater. Interfaces.

[cit31] Leblebici S. Y., Chen T. L., Olalde-Velasco P., Yang W., Ma B. (2013). ACS Appl. Mater. Interfaces.

[cit32] Zhong Y., Tada A., Izawa S., Hashimoto K., Tajima K. (2014). Adv. Energy Mater..

[cit33] Intemann J. J., Yao K., Ding F., Xu Y., Xin X., Li X., Jen A. K. Y. (2015). Adv. Funct. Mater..

[cit34] Randazzo J. M., Marante C., Chattopadhyay S., Schneider B. I., Olsen J., Argenti L. (2023). Phys. Rev. Res..

[cit35] Hsu C.-P. (2020). Phys. Chem. Chem. Phys..

[cit36] Vehmanen V., Tkachenko N. V., Imahori H., Fukuzumi S., Lemmetyinen H. (2001). Spectrochim. Acta, Part A.

[cit37] Sugahara T., Hashizume D., Espinosa Ferao A., Masada K., Tokitoh N., Sasamori T. (2025). Angew. Chem., Int. Ed..

[cit38] Shibutani Y., Kusumoto S., Nozaki K. (2024). Chem. Sci..

[cit39] Yang M., Yin B., Hu G., Cao Y., Lu S., Chen Y., He Y., Yang X., Huang B., Li J., Wu B., Pang S., Shen L., Liang Y., Wu H., Lan L., Yu G., Huang F., Cao Y., Duan C. (2024). Chem.

[cit40] Yokoyama S., Utsunomiya S., Seo T., Saeki A., Ie Y. (2024). Adv Sci.

[cit41] Wang H., Dai X., Liao C., Xu X., Peng Q. (2025). Chem. Commun..

[cit42] Wang Y., Yang M., Chen Z., Zhong J., Zhao F., Wei W., Yuan X., Zhang W., Ma Z., He Z., Liu Z., Huang F., Cao Y., Duan C. (2025). Nat. Commun..

[cit43] Wu Y., Yuan Y., Sorbelli D., Cheng C., Michalek L., Cheng H.-W., Jindal V., Zhang S., LeCroy G., Gomez E. D., Milner S. T., Salleo A., Galli G., Asbury J. B., Toney M. F., Bao Z. (2024). Nat. Commun..

[cit44] An K., Zhong W., Peng F., Deng W., Shang Y., Quan H., Qiu H., Wang C., Liu F., Wu H., Li N., Huang F., Ying L. (2023). Nat. Commun..

[cit45] Peng J., Ma L., Li H., Wang G., Chen Z., Chen F., Hou J., Zhang S. (2024). Mater. Chem. Front..

[cit46] Li C., Yao G., Gu X., Lv J., Hou Y., Lin Q., Yu N., Abbasi M. S., Zhang X., Zhang J., Tang Z., Peng Q., Zhang C., Cai Y., Huang H. (2024). Nat. Commun..

[cit47] Gu X., Wei Y., Zeng R., Lv J., Hou Y., Yu N., Tan S., Wang Z., Li C., Tang Z., Peng Q., Liu F., Cai Y., Zhang X., Huang H. (2025). Angew. Chem., Int. Ed..

[cit48] Schubert Y., Luber S., Marzari N., Linscott E. (2024). npj Comput. Mater..

[cit49] Chen Y., Zheng Y., Wang J., Zhao X., Liu G., Lin Y., Yang Y., Wang L., Tang Z., Wang Y., Fang Y., Zhang W., Zhu X. (2024). Sci. Adv..

[cit50] Sangwan V. K., Martin Z., Li G., Qin F., Hadke S., Pankow R. M., Jeon W. C., Zheng D., Cho Y., Young R. M., Kohlstedt K. L., Wasielewski M. R., Schatz G. C., Facchetti A., Hersam M. C., Marks T. J. (2024). J. Mater. Chem. A.

[cit51] Song Y., Cao S., Wang Y., Chen M., Zhang Y., Li Q., Han S., Liang Y., Cai L., Zhao J., Zou B. (2025). Light Sci. Appl..

[cit52] Xiao Y., Cui Y., Yuan H., Wang J., Chen Z., Wang G., Fu W., Fu Z., Wang Y., Zhang T., Yu Y., Yu R., Zuo G., Zhang M., Hao X., Hou J. (2025). Energy Environ. Sci..

[cit53] Li B., Murto P., Chowdhury R., Brown L., Han Y., Londi G., Beljonne D., Bronstein H., Friend R. H. (2026). Nat. Mater..

[cit54] Lv G., Yu X., Wang J., Qiu J., Yang D., Zhu Y. (2025). Adv. Funct. Mater..

[cit55] Lu T. (2025). Angew. Chem., Int. Ed..

[cit56] Xie Z., Liu D., Gao C., Zhang X., Dong H., Hu W. (2025). J. Am. Chem. Soc..

[cit57] Zhang L., Zhao Y., Li J., Fu Y., Peng B., Yang J., Lu X., Miao Q. (2025). J. Am. Chem. Soc..

[cit58] Fowles D. J., Connaughton B. J., Carter J. W., Mitchell J. B. O., Palmer D. S. (2025). Chem. Rev..

[cit59] Chen X., Tian Y., Wupur A., Chen T., Li S., Zhang N., Liu H., Lu X., Shi M., Chen H. (2025). Angew. Chem., Int. Ed..

[cit60] Xu R., Jiang Y., Lei H., Liu F., Liu K., Feng L., Ran G., Zhang W., Zhong C., Zhu X. (2025). Sci. Adv..

[cit61] Zhang M., Zhu L., Yan J., Xue X., Wang Z., Eisner F., Zhou G., Zeng R., Kan L., Wu L., Zhong W., Zhang A., Han F., Song J., Hartmann N., Zhou Z., Jing H., Zhu H., Xu S., Nelson J., Zhang Y., Liu F. (2025). Joule.

[cit62] Deng B., Li Y., Lu Z., Zheng K., Xu T., Wang S., Luo X., Grandidier B., Zhang J., Zhu F. (2025). Nat. Commun..

[cit63] Choi Y., Kim D., Ryu S. U., Kim H., Kim S., Kim M., Park T. (2025). Adv. Energy Mater..

[cit64] Lu Y., Gao J. (2025). Wiley Interdiscip. Rev.:Comput. Mol. Sci..

[cit65] Zhu L., Huang M., Han G., Wei Z., Yi Y. (2025). Angew. Chem., Int. Ed..

[cit66] Khan Z., Alathlawi H. J., Salem K. H., Naqvi S. M. K. A., Jaghdam I. H., Soliman M. S., Hasanin T. H. A., Ali S., Bhatti H. N., Khera R. A. (2025). RSC Adv..

[cit67] Yu J., Pu J., Xie D., Ai Z., Lang Y., Cao M., Duan C., Lu L., Li G. (2025). Nat. Commun..

