# Supplementary material for: Terminal acceptor engineering for reduced energy dissipation and enhanced charge transport in benzodithiophene-core based donor molecules: a computational route to efficient organic solar cells
Source: Nanoscale Adv. 2026 Apr 23;8(10):3200–12. doi: 10.1039/d5na01002k (PMC13104626; doi:10.1039/d5na01002k)
Supplement: NA-008-D5NA01002K-s001 [file NA-008-D5NA01002K-s001.pdf]

Supplementary Information (SI) for Nanoscale Advances.
This journal is © The Royal Society of Chemistry 2026

Terminal Acceptor Engineering for Reduced Energy Dissipation and Enhanced Charge Transport in Benzodithiophene-Core Based Donor Molecules: A Computational Route to Efficient Organic Solar Cells

Sabiha Khanam,[a] Zahraa Falah Khudair,[b] Ines Hilali Jaghdam,[c] Rana Farhat Mehmood,[d] Muhammad Imran,[e] Mohamed S. Soliman,[f] Ahmed M. Shawky,[g] Rabaa Bousbih,[h] Syed Muhammad Kazim Abbas Naqvi,*[i,j] and Rasheed Ahmad Khera*[a]

Theoretical Methods

Four hybrid functionals namely B3LYP,[1] CAM-B3LYP,[2] MPW1PW91,[3] and wB97XD[4] were initially tested with the 6-31G(d,p) basis set for ground state optimizations and molar absorptivity calculations of the reference molecule.[5] The optimized system exhibited λ_{max} values of 661 nm (B3LYP), 453 nm (CAM-B3LYP), 604 nm (MPW1PW91) and 419 nm (WB97XD). Comparison with the experimental λ_{max} 696 nm revealed deviations of 35 nm, 243 nm, 92 nm and 277 nm respectively, confirming B3LYP/6-31G(d,p) exhibiting a difference of 35 nm emerged as the most suitable functional. Although long-range corrected functionals are often recommended for charge transfer excitations, B3LYP has been widely employed in donor-acceptor organic semiconductors where the excitation has mixed local and ICT character. In the present study, B3LYP was selected based on benchmarking against available experimental absorption data, where it provided the closest agreement for the reference molecule. Moreover, the focus of this work is on relative trends across a homologous molecular series rather than absolute excitation energies, for which B3LYP is known to be reliable. Nevertheless, possible underestimation of long-range charge transfer excitation energies is acknowledged as a limitation and does not affect the comparative conclusions drawn in this study. The graphical representation of λ_{max} is shown in **Fig. S1**. DOS analysis was conducted using PyMOlyze 1.1[6] to probe the contributions of donor and acceptor fragments to the absorption features. The transition density matrix (TDM)[7] maps were generated with Multiwfn 3.8[8] to visualize the nature of electronic excitations. Reorganization energies (λ) were computed at the B3LYP/6-31G(d,p) level, separating internal and external contributions.[9,10] Electron (λ_e) and hole (λ_h) reorganization energies were calculated using the Marcus theory equations:

$$\lambda_e = \left[E_{\circ}^{-} - E_{-}\right] + \left[E_{-}^{\circ} - E_{\circ}\right] \qquad \text{(S1)}$$

$$\lambda_h = \left[E_{\circ}^{+} - E_{+}\right] + \left[E_{+}^{\circ} - E_{\circ}\right] \qquad \text{(S2)}$$

λ_e and λ_h is the reorganization energy of an electron and hole, respectively. $E_\circ$ is neutral ground state energy, E_- and E_+ correspond to optimized anionic and cationic total energies. E_-° and E_+° are neutral energies computed at optimized anionic and cationic geometries. $E_\circ^-$ and $E_\circ^+$ are single point energies of the anion and cation at the neutral geometry.

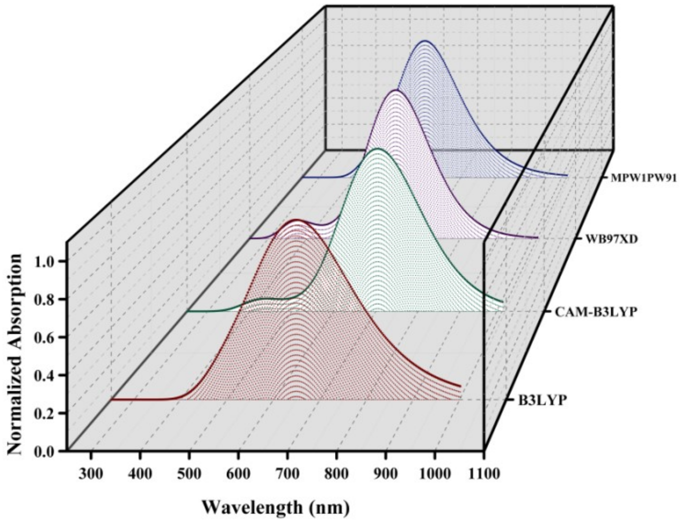

Fig. S1 Comparison of four distinct functionals (B3LYP, CAMB3LYP, WB97XD, and MPW1PW91) using 6-31G(d,p) level of theory

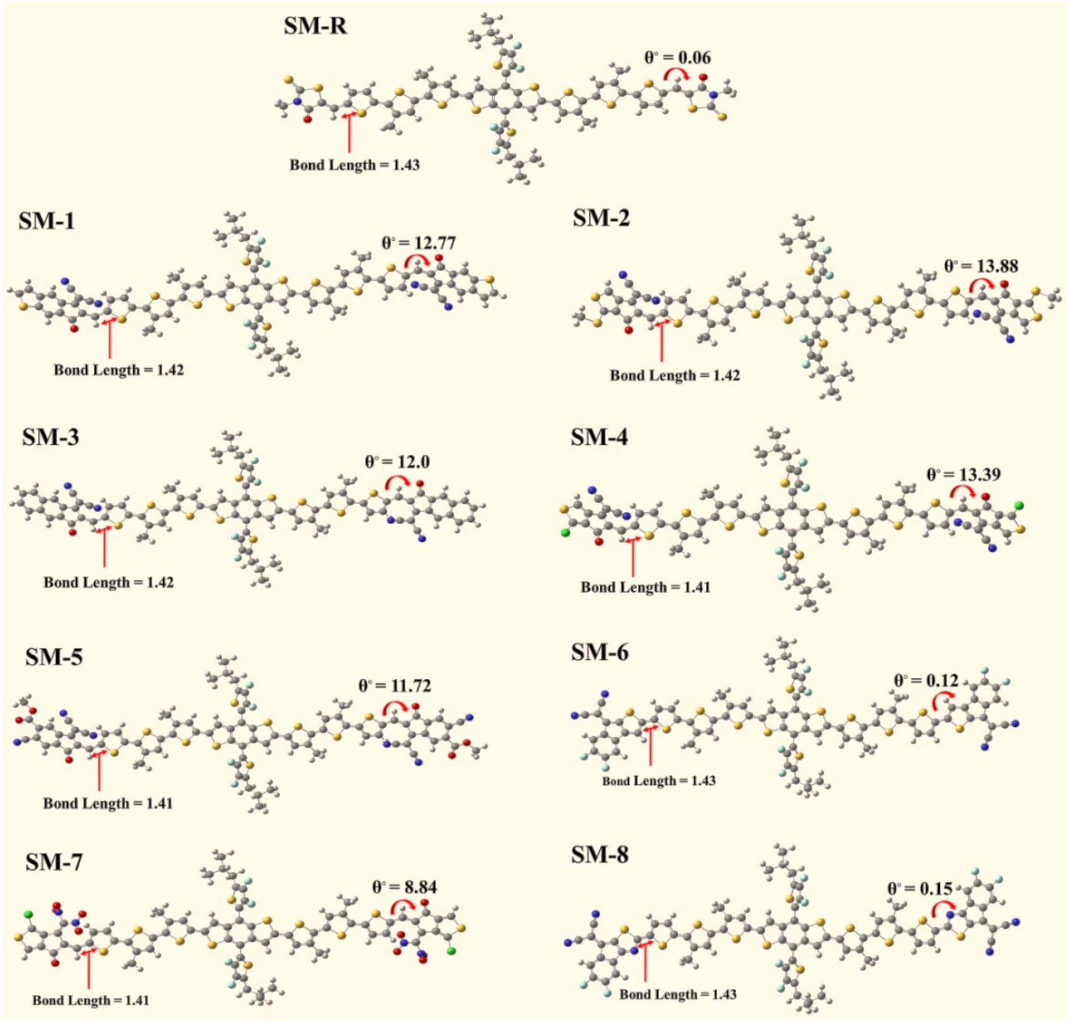

Fig. S2 Optimized geometries of reference **SM-R** and all designed molecules

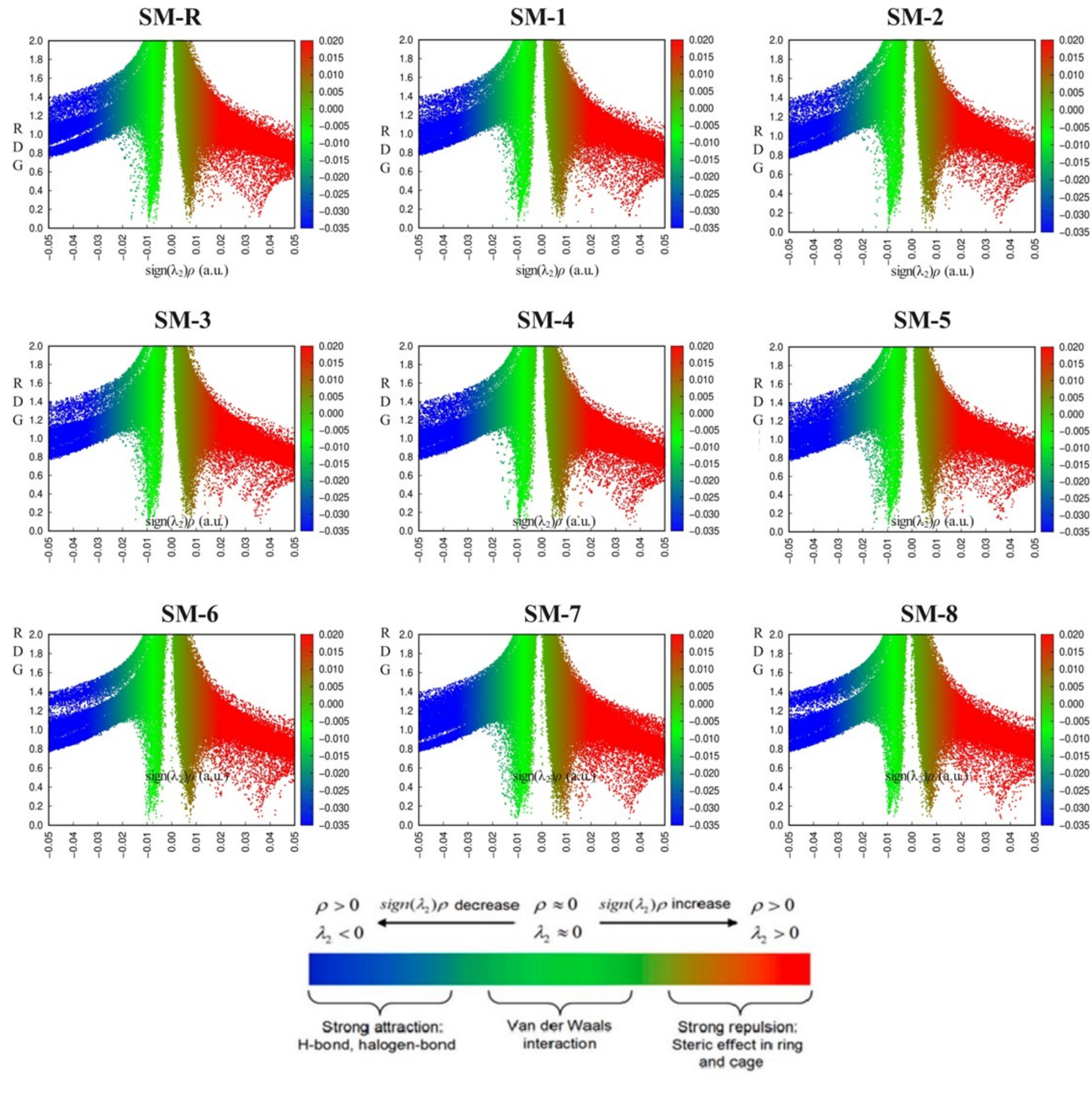

Fig. S3 Reduced density gradient plots for the reference and designed donor molecules

Table S1 Percent contributions of the acceptor, π-bridges, and donor core to the HOMO and LUMO for **SM-R** and designed molecules

Molecules	Excitation Energy States	Percentage contribution of Acceptor (%)	Percentage contribution of Bridge (%)	Percentage contribution of Donor core (%)
**SM-R**	HOMO	9.6	62.5	27.8
	LUMO	37.1	51.0	11.9
**SM-1**	HOMO	9.5	60.6	29.9
	LUMO	55.7	39.4	4.9
**SM-2**	HOMO	10.0	60.3	29.8
	LUMO	57.2	38.3	4.5
**SM-3**	HOMO	9.9	60.2	30.0
	LUMO	57.8	37.8	4.4
**SM-4**	HOMO	9.7	58.9	31.4
	LUMO	57.2	58.8	4.0
**SM-5**	HOMO	9.1	57.1	33.8
	LUMO	61.9	34.9	3.1
**SM-6**	HOMO	11.8	61.7	26.5
	LUMO	84.2	14.8	1.0
**SM-7**	HOMO	10.1	57.0	32.9
	LUMO	65.1	32.3	2.7
**SM-8**	HOMO	10.5	61.0	28.6
	LUMO	83.8	15.1	1.0

Table S2 Calculated dipole moments of the reference and designed donor molecules in gas and solvent phases

Molecules	Dipole moment in gas phase (D)	Dipole moment in the solvent phase (D)
**SM-R**	2.2298	2.7584
**SM-1**	5.7502	5.7849
**SM-2**	5.5855	6.5461
**SM-3**	4.7919	4.5499
**SM-4**	2.6553	3.0861
**SM-5**	6.7676	8.2140
**SM-6**	4.9285	6.9554
**SM-7**	3.6711	11.6833
**SM-8**	9.2658	6.0512

Table S3 Calculated optical absorption parameters of the reference and designed donor molecules in the gas phase

Molecules	Calculated λ_{max} (nm)	Excitation energies (E_x) (eV)	Oscillator strength (f)
**SM-R**	629	1.97	3.70
**SM-1**	809	1.53	1.71
**SM-2**	715	1.73	3.05
**SM-3**	708	1.74	3.21
**SM-4**	732	1.69	2.86
**SM-5**	758	1.63	2.58
**SM-6**	826	1.49	1.65
**SM-7**	722	1.71	3.17
**SM-8**	797	1.55	2.31

Table S4 Calculated optical absorption parameters of the reference and designed donor molecules in the solvent phase

Molecules	Exp. λ_{max} (nm)	Calculated λ_{max} (nm)	Excitation energies (E_x) (eV)	Oscillator strength (f)
**SM-R**	696	661	1.87	3.99
**SM-1**	-	756	1.63	3.42
**SM-2**	-	766	1.61	3.28
**SM-3**	-	774	1.60	3.38
**SM-4**	-	785	1.57	3.18
**SM-5**	-	827	1.49	2.59
**SM-6**	-	874	1.41	2.04
**SM-7**	-	898	1.38	2.43
**SM-8**	-	903	1.37	1.90

Table S5 Calculated light-harvesting efficiency (LHE) and excited state lifetime (τ) of the reference and designed donor molecules

Molecules	LHE	τ (ns)
**SM-R**	0.9998	0.107
**SM-1**	0.9996	0.165
**SM-2**	0.9995	0.176
**SM-3**	0.9996	0.173
**SM-4**	0.9993	0.191
**SM-5**	0.9974	0.260
**SM-6**	0.9909	0.369
**SM-7**	0.9963	0.324
**SM-8**	0.9874	0.420

References

J. Tirado-Rives and W. L. Jorgensen, *J Chem Theory Comput*, 2008, **4**, 297–306.

T. Yanai, D. P. Tew and N. C. Handy, *Chem Phys Lett*, 2004, **393**, 51–57.

Y. Zhao, J. Pu, B. J. Lynch and D. G. Truhlar, *Phys Chem Chem Phys*, 2004, **6**, 673–676.

H. Fang and Y. Kim, *J Chem Theory Comput*, 2011, **7**, 642–657.

S. M. Kazim Abbas Naqvi, F. Abbas, S. Bibi, M. K. Shehzad, N. Alhokbany, Y. Zhu, H. Long, R. B. Vasiliev, Z. Nazir and S. Chang, *RSC Adv*, 2024, **14**, 29942–29954.

N. M. O'boyle, A. L. Tenderholt and K. M. Langner, *J Comput Chem*, 2008, **29**, 839–845.

J. M. Randazzo, C. Marante, S. Chattopadhyay, B. I. Schneider, J. Olsen and L. Argenti, *Phys Rev Res*, 2023, **5**, 043115.

T. Lu and F. Chen, *J Comput Chem*, 2012, **33**, 580–592.

C.-P. Hsu, *Phys Chem Chem Phys*, 2020, **22**, 21630–21641.

V. Vehmanen, N. V. Tkachenko, H. Imahori, S. Fukuzumi and H. Lemmetyinen, *Spectrochim Acta Part A Mol Biomol Spectrosc*, 2001, **57**, 2229–2244.
