# Supplementary figures and images for: Terminal acceptor engineering for reduced energy dissipation and enhanced charge transport in benzodithiophene-core based donor molecules: a computational route to efficient organic solar cells
Source: Nanoscale Adv. 2026 Apr 23;8(10):3200–12. doi: 10.1039/d5na01002k (PMC13104626; doi:10.1039/d5na01002k)

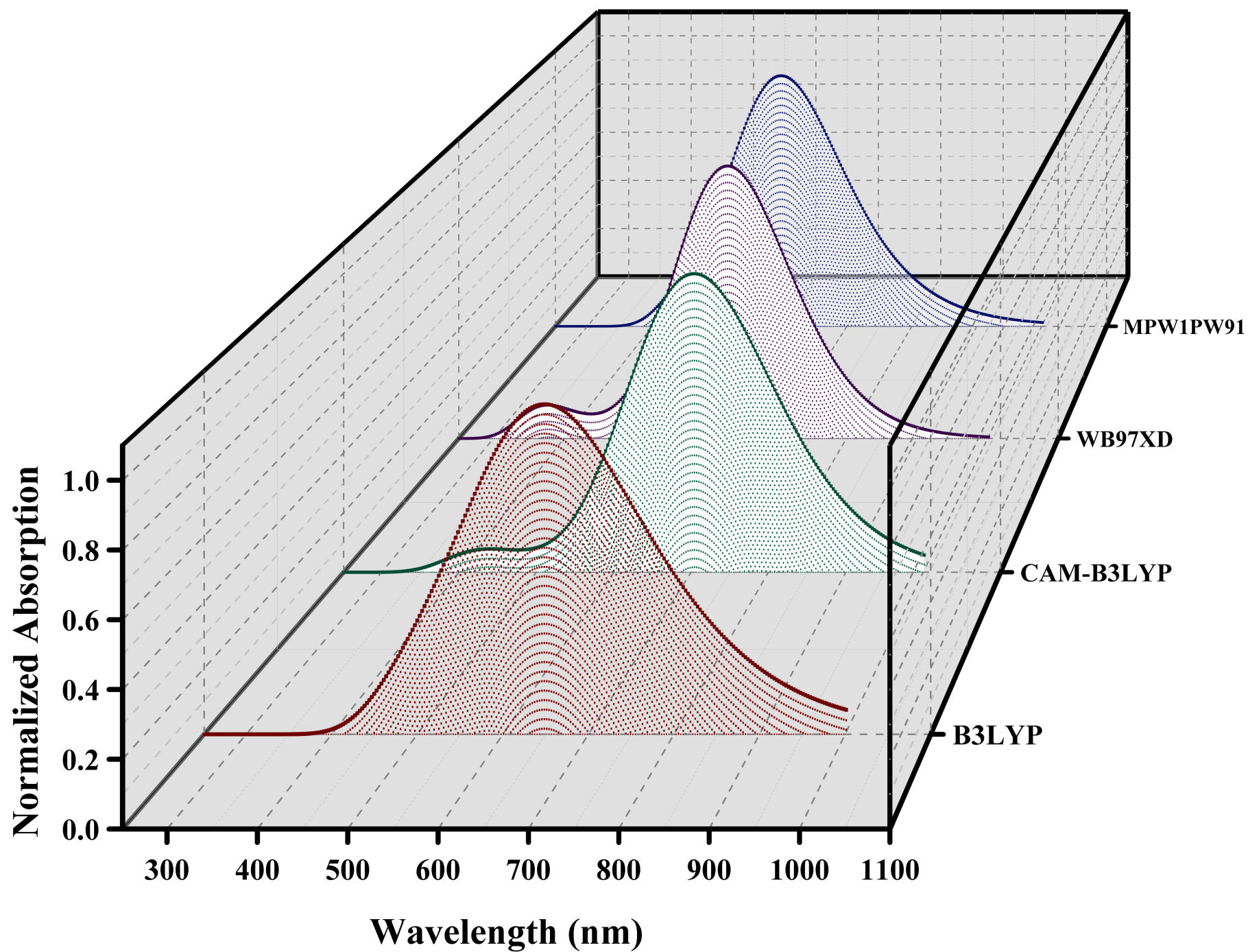

Supplement: NA-008-D5NA01002K-s003 [file NA-008-D5NA01002K-s003.pdf]

**SM-R**

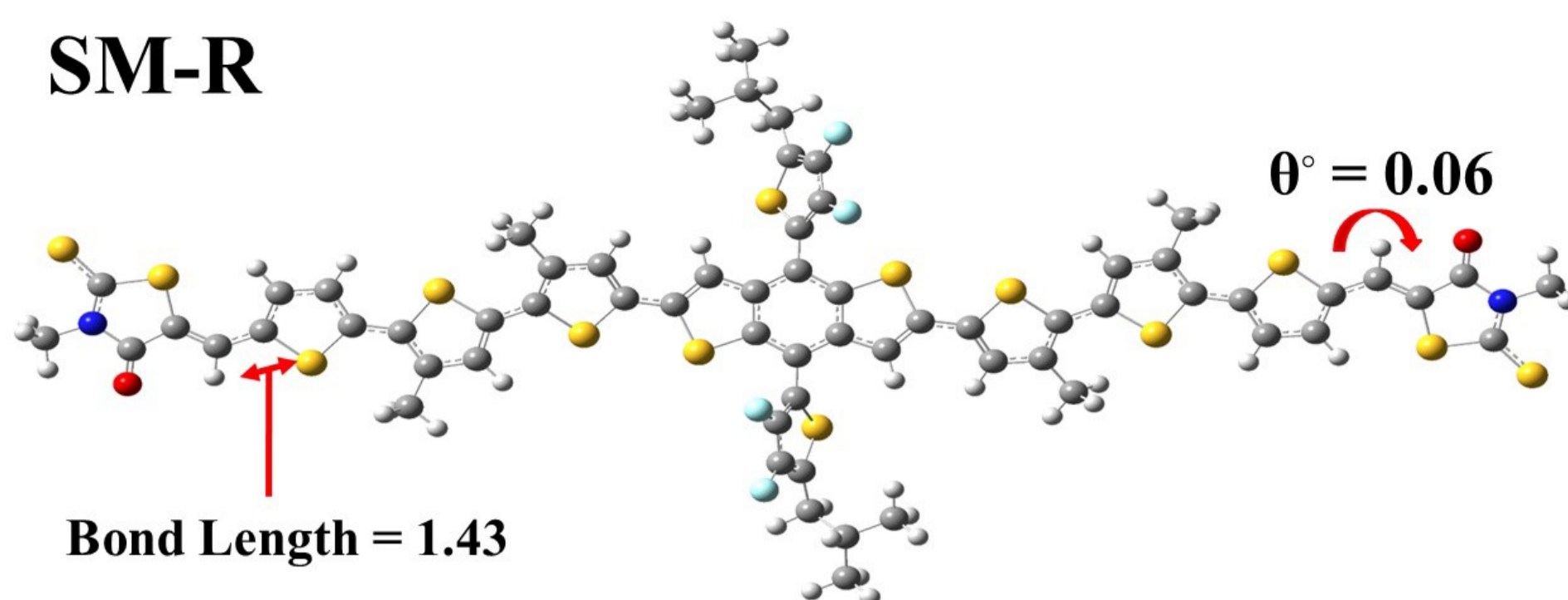

**SM-A1**

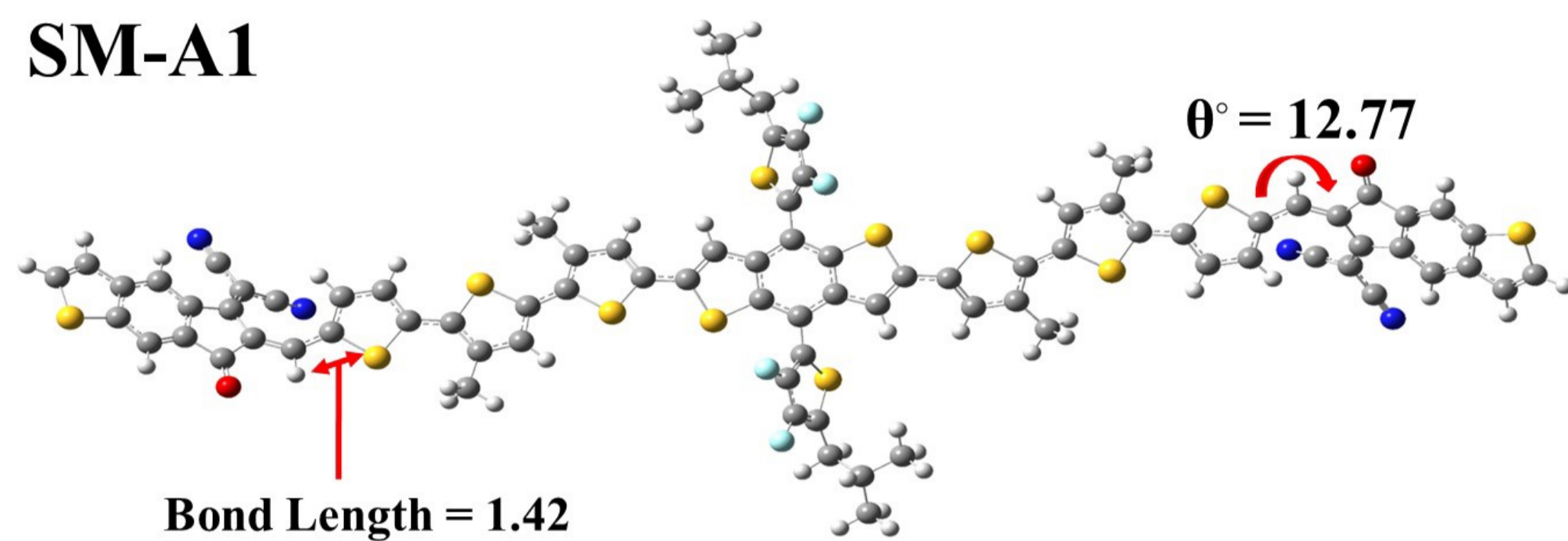

**SM-A2**

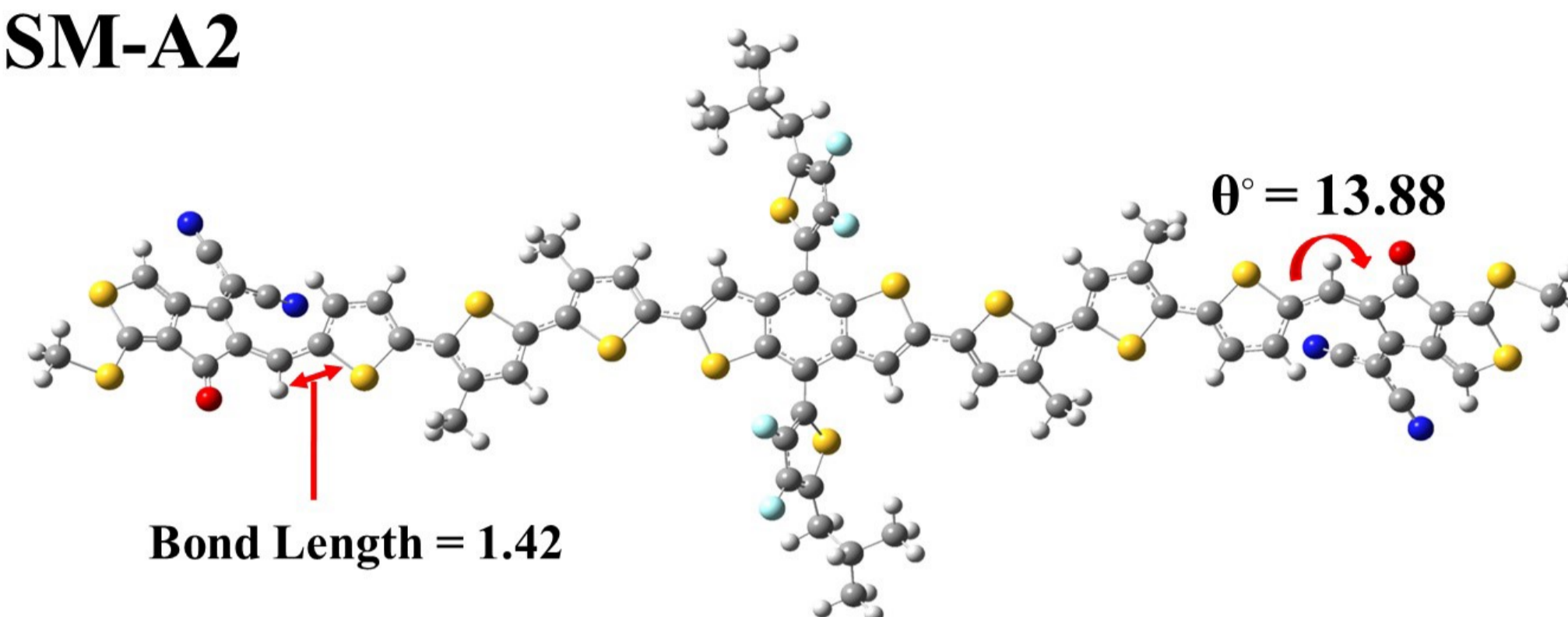

**SM-A3**

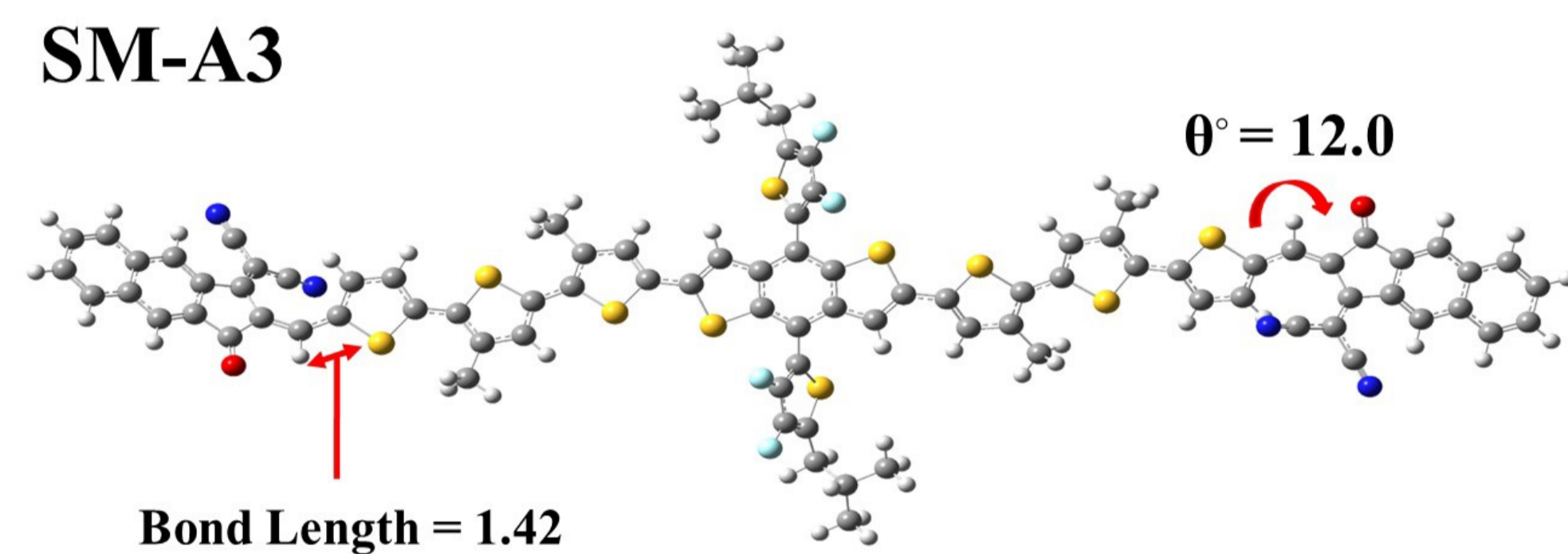

**SM-A4**

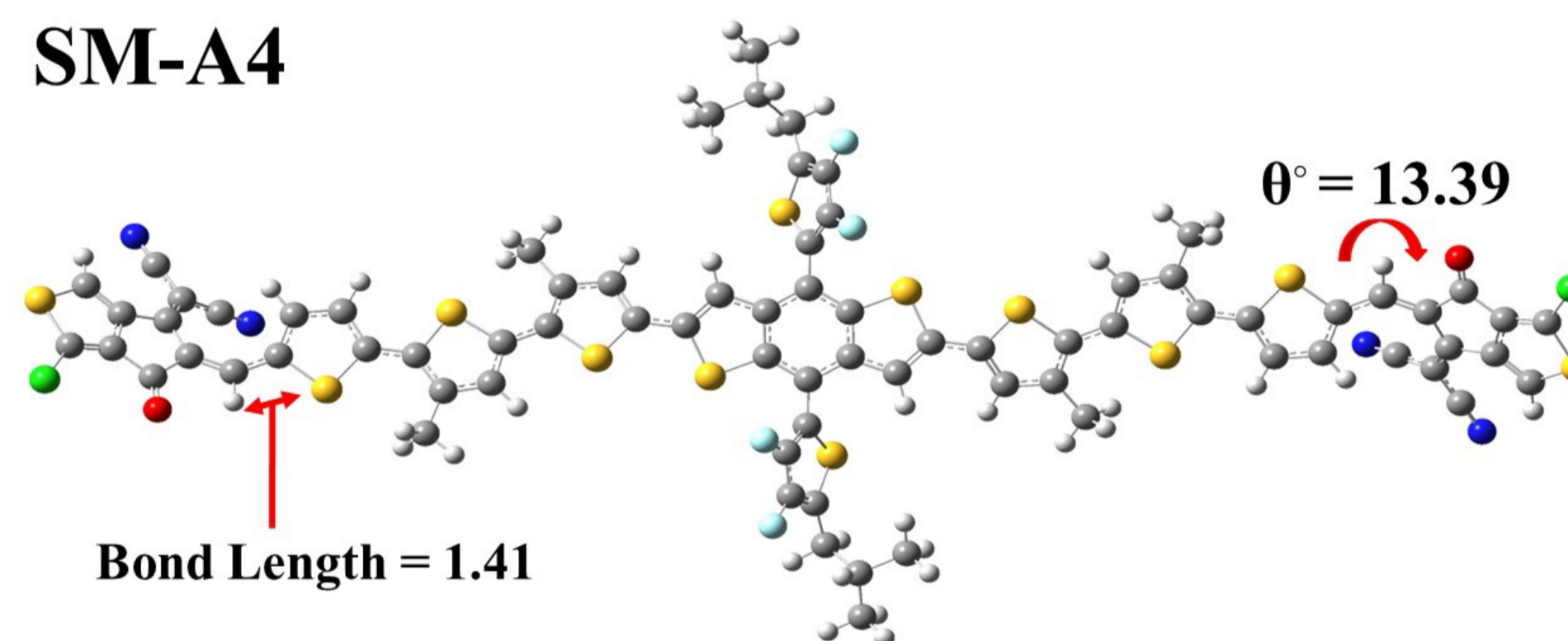

**SM-A5**

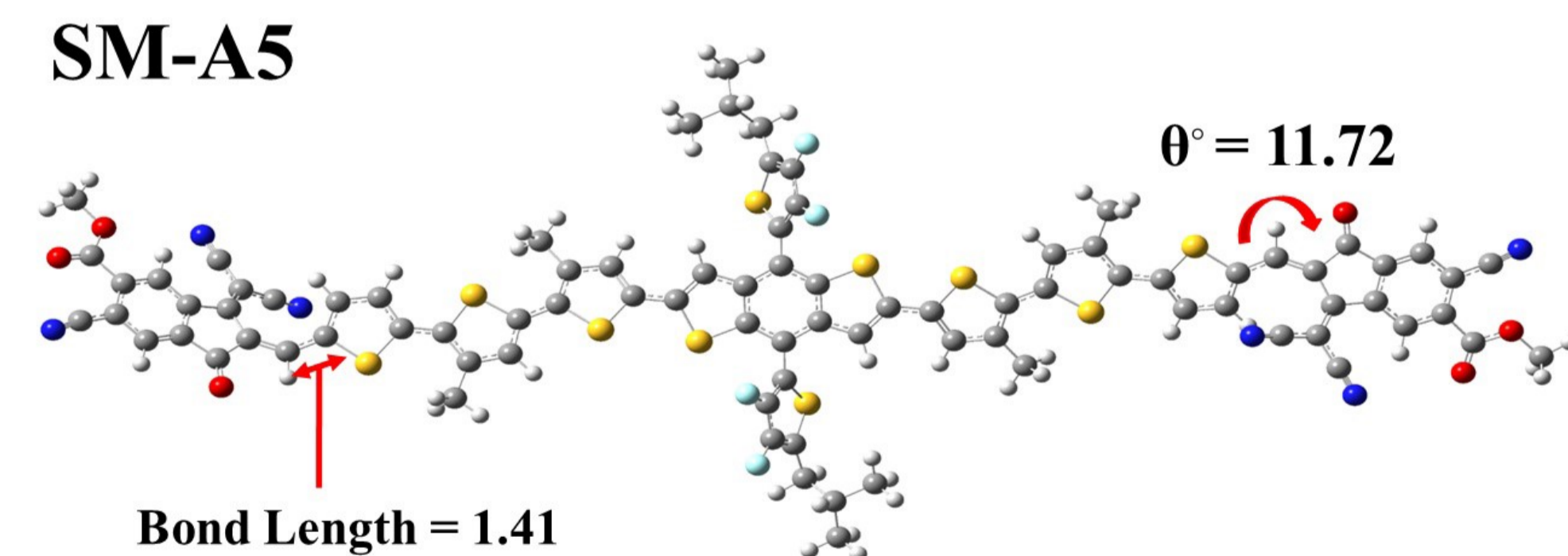

**SM-A6**

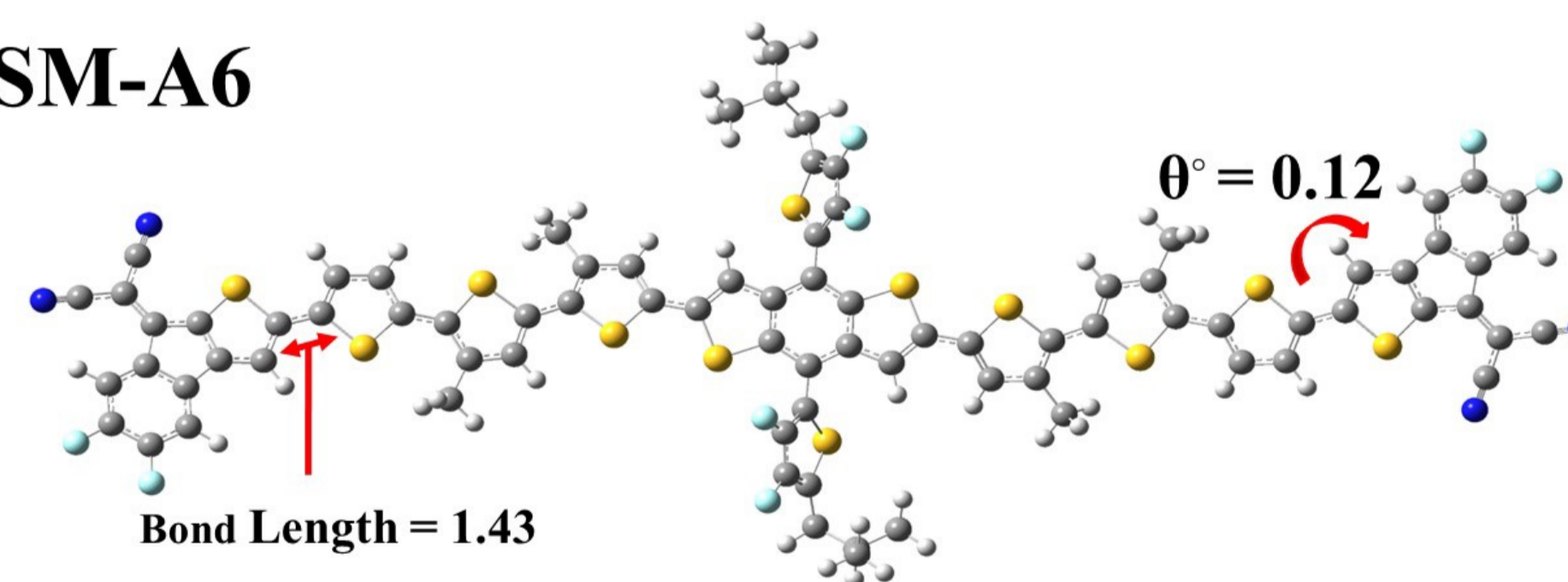

**SM-A7**

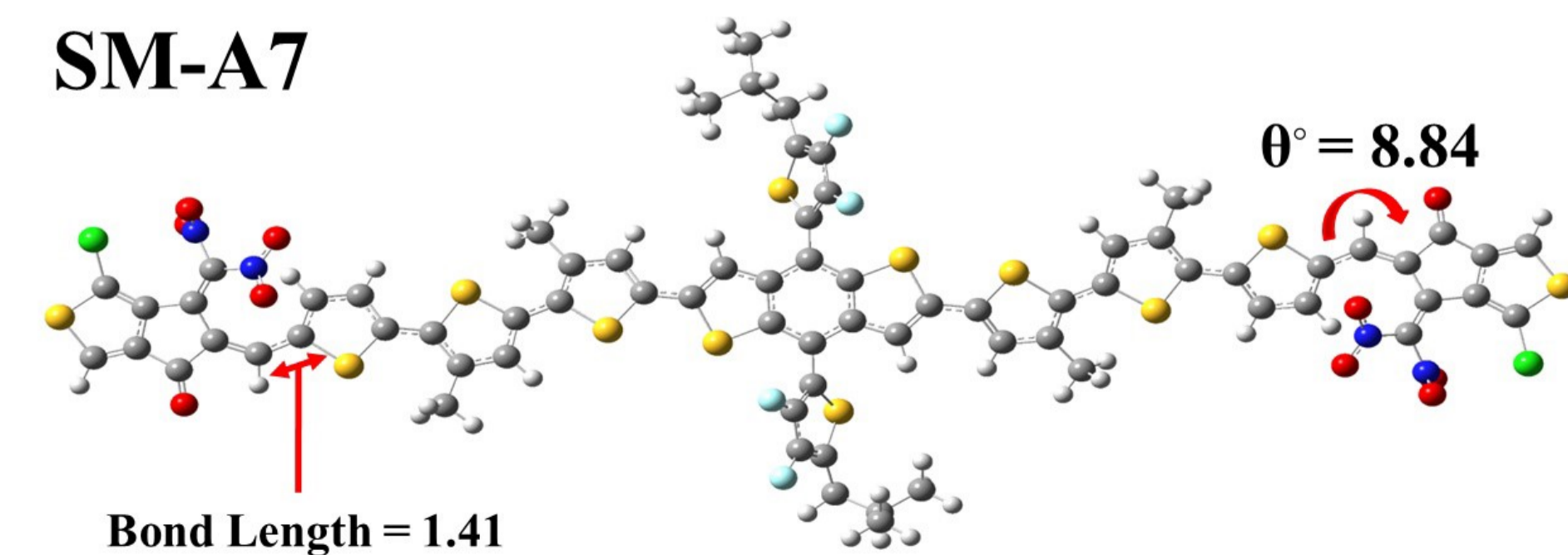

**SM-A8**

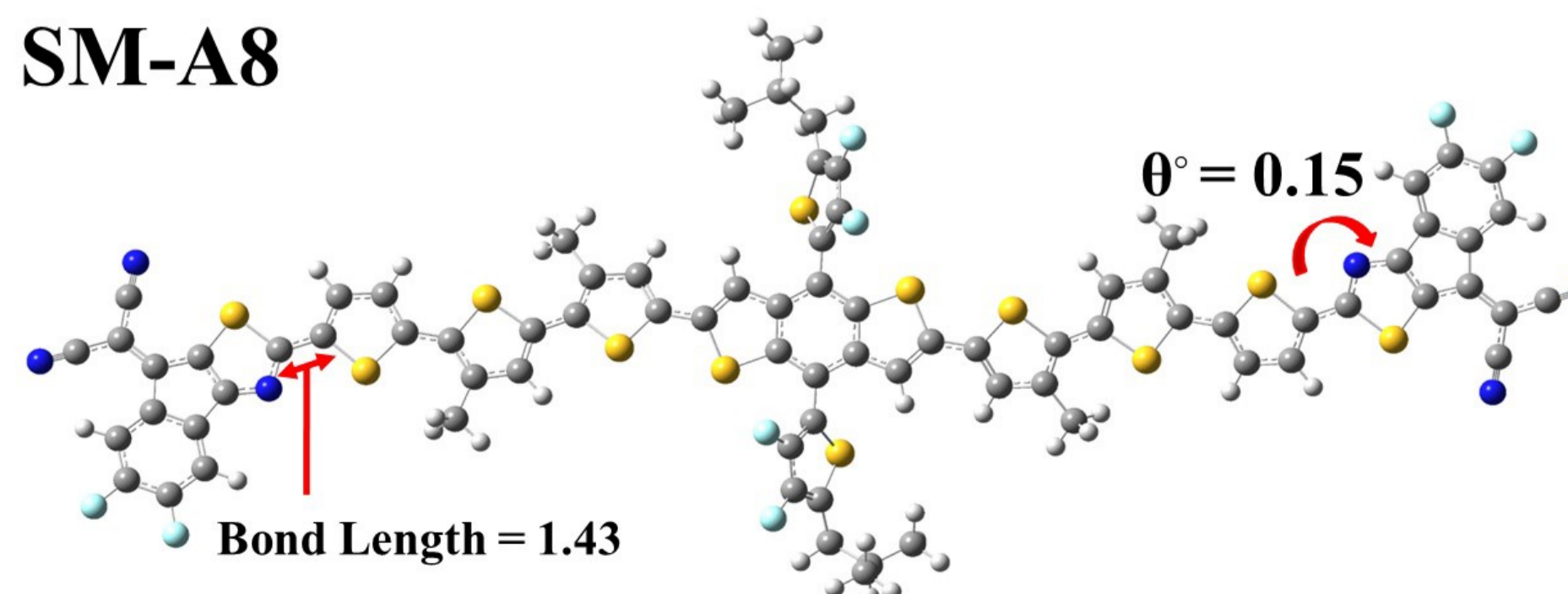

Supplement: NA-008-D5NA01002K-s004 [file NA-008-D5NA01002K-s004.pdf]

SM-R

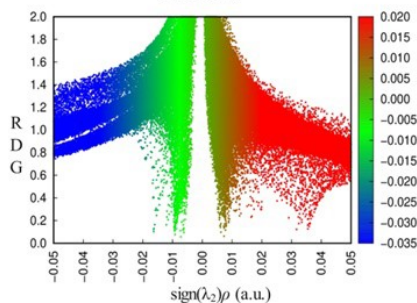

SM-1

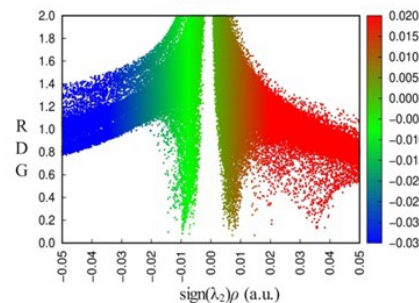

SM-2

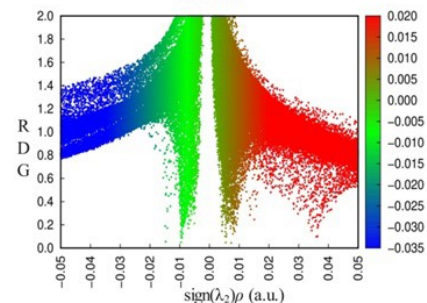

SM-3

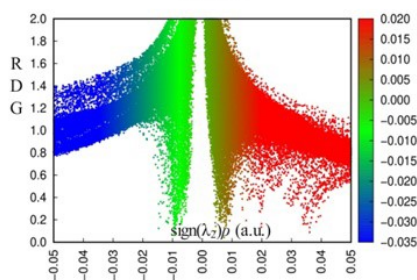

SM-4

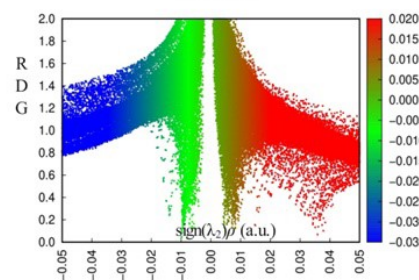

SM-5

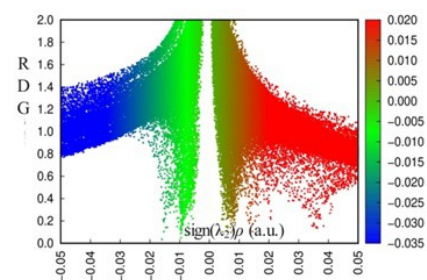

SM-6

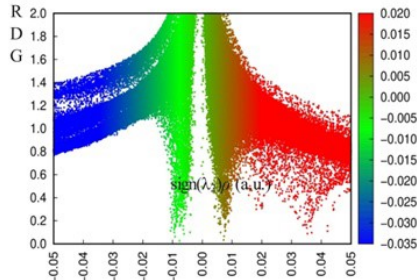

SM-7

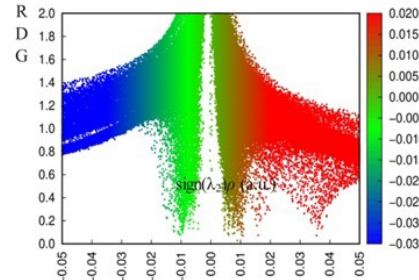

SM-8

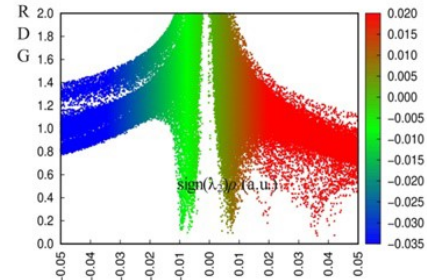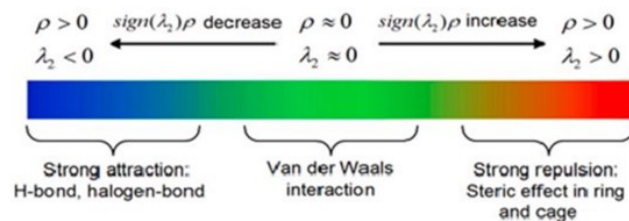

Supplement: NA-008-D5NA01002K-s005 [file NA-008-D5NA01002K-s005.pdf]
